# Backbone Protecting Groups for Enhanced Peptide and Protein Synthesis

**DOI:** 10.1002/anie.202509939

**Published:** 2025-07-16

**Authors:** Samuel J. Paravizzini, Linda M. Haugaard‐Kedström, Craig A. Hutton, John A. Karas

**Affiliations:** ^1^ School of Chemistry The University of Melbourne Grattan Street Parkville Victoria 3010 Australia; ^2^ PolyPeptide Laboratories (Sweden) AB Limhamnsvägen 108, PO Box 30089 Limhamn 20061 Sweden; ^3^ The Florey 30 Royal Parade Parkville Victoria 3052 Australia

**Keywords:** Amides, Chemical protein synthesis, Peptides, Protecting groups, Solid‐phase peptide synthesis

## Abstract

Solid‐phase peptide synthesis has become an indispensable technique for the routine preparation of linear peptides of up to approximately 40 amino acids in length. However, the solid‐phase approach is still hampered by chain insolubility and aggregation, which reduces synthetic yields. Moreover, many of the deletion impurities that can form are often chromatographically inseparable from the target sequence, which diminishes final product purity. The use of backbone *N*‐protecting groups can ameliorate this synthetic inefficiency by increasing peptide chain solubility and suppressing aggregation. Backbone protection is also useful for promoting peptide macrocyclization, suppressing common side reactions in peptide chemistry, and improving solution‐phase handling. Commercially available precursors containing benzyl‐based groups and pseudoprolines have found widespread use, in academic laboratories and industry. A range of other strategies have also been investigated in a bid to increase the utility of backbone protecting groups and to develop more efficient methods for their introduction and removal. This review provides a comprehensive account of the state of the art, and includes detailed synthetic methods relating to the use of backbone protection, and its application to “difficult” peptides and proteins of biological significance. The strengths and weaknesses of each approach are analyzed, and a commentary on future directions is provided.

## Graphical abstract

This review provides a comprehensive account of the use of backbone *N*‐protecting groups in peptide synthesis. It includes detailed synthetic methods relating to their introduction and removal and their application to “difficult” peptides and proteins of biological significance in both academic and industry contexts.

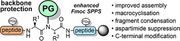

## 1 Introduction

**Scheme 1:**
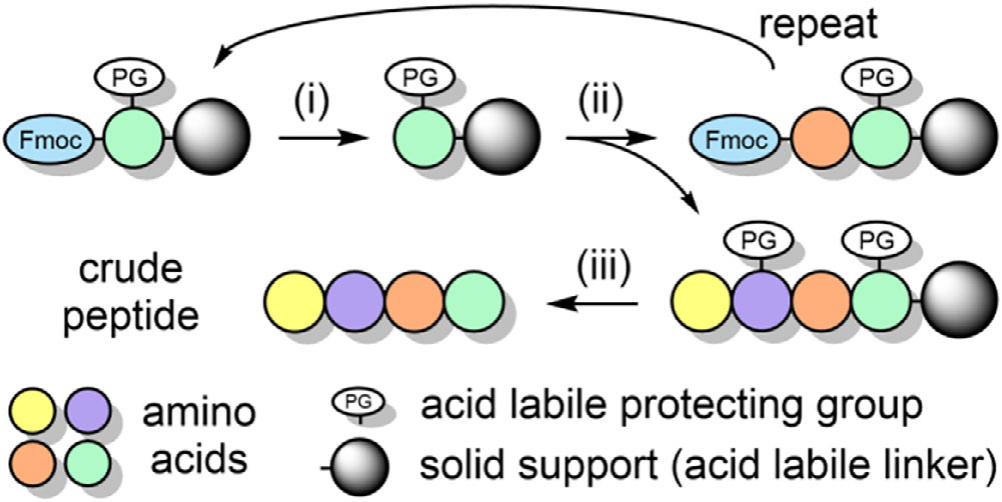
The iterative process of Fmoc SPPS. i) 20% piperidine in *N*,*N*‐dimethylformamide (DMF). ii) Fmoc‐amino acid and coupling reagents. iii) TFA.

9‐Fluorenylmethyloxycarbonyl (Fmoc) solid‐phase peptide synthesis (SPPS) is the gold standard method for the routine and rapid preparation of linear peptides <40 residues in length.^[^ ^1^, ^2^, ^3^ ^]^ It involves the covalent attachment of the C‐terminal amino acid of the peptide onto insoluble resin beads (typically consisting of polystyrene), followed by assembly via iterative coupling/deprotection cycles with Fmoc‐amino acids. The peptide is then cleaved from the resin (and globally deprotected) with trifluoroacetic acid (TFA) and purified by reversed‐phase chromatography (Scheme 1). Peptides bearing macrocycles,^[^ ^4^, ^5^, ^6^ ^]^ post‐translational modifications (PTMs),^[^ ^7^, ^8^, ^9^, ^10^, ^11^, ^12^ ^]^ non‐native substitutions (e.g., d‐amino acids),^[^ ^13^, ^14^, ^15^, ^16^ ^]^ fluorescent probes,^[^ ^17^, ^18^ ^]^ and isotopic labels^[^ ^19^, ^20^, ^21^ ^]^ are now prepared routinely via solid‐phase methods. Peptide libraries can also be rapidly assembled, for epitope mapping^[^ ^22^, ^23^ ^]^ and structure–activity relationship studies.^[^ ^24^ ^]^ Native chemical ligation (NCL) is underpinned by SPPS,^[^ ^25^ ^]^ whereby short synthetic peptides—typically 20–40 amino acids in length—are linked together to form a larger protein of interest.^[^ ^26^, ^27^, ^28^, ^29^ ^]^ Moreover, solid‐phase methods enable single‐shot protein assembly,^[^ ^30^ ^]^ with the current benchmark being 214 amino acids in length.^[^ ^31^ ^]^ Many peptide‐based active pharmaceutical ingredients (APIs)—such as the 36‐residue HIV fusion inhibitor enfuvirtide^[^ ^32^ ^]^ and the GLP‐1 agonist tirzepatide^[^ ^33^, ^34^ ^]^—are now produced via large‐scale SPPS (in combination with solution‐phase fragment condensations).

Peptide therapeutics are becoming increasingly popular due to their high target selectivity, high potency, and excellent safety profile.^[^ ^35^, ^36^ ^]^ The demand for peptide APIs is projected to increase significantly:^[^ ^36^, ^37^ ^]^ the 2023 annual market value was USD 43 billion and is expected to rise to USD 87 billion by 2032.^[^ ^38^ ^]^ There are more than 60 FDA‐approved peptide therapeutics with a further 150 in clinical development,^[^ ^36^, ^39^, ^40^ ^]^ to treat a range of diseases.^[^ ^41^, ^42^, ^43^ ^]^ Notable examples include buserelin for the treatment of prostate cancer and endometriosis,^[^ ^44^ ^]^ and GLP‐1 agonists such as semaglutide for the treatment of diabetes^[^ ^45^ ^]^ and obesity.^[^ ^46^ ^]^ Self‐assembling peptide‐based nanomaterials have also reached the clinic,^[^ ^47^, ^48^ ^]^ for applications in regenerative medicine and wound healing.^[^ ^49^, ^50^ ^]^ Chemical methods are fast becoming competitive with biosynthetic approaches for producing larger peptides due to heat‐assisted SPPS,^[^ ^51^ ^]^ reduced solvent usage,^[^ ^52^ ^]^ and the use of greener solvents.^[^ ^53^, ^54^ ^]^ However, to meet future demand, further improvements in SPPS methodology are highly sought after to expedite peptide drug discovery and improve peptide API manufacturing.

**Figure 1:**
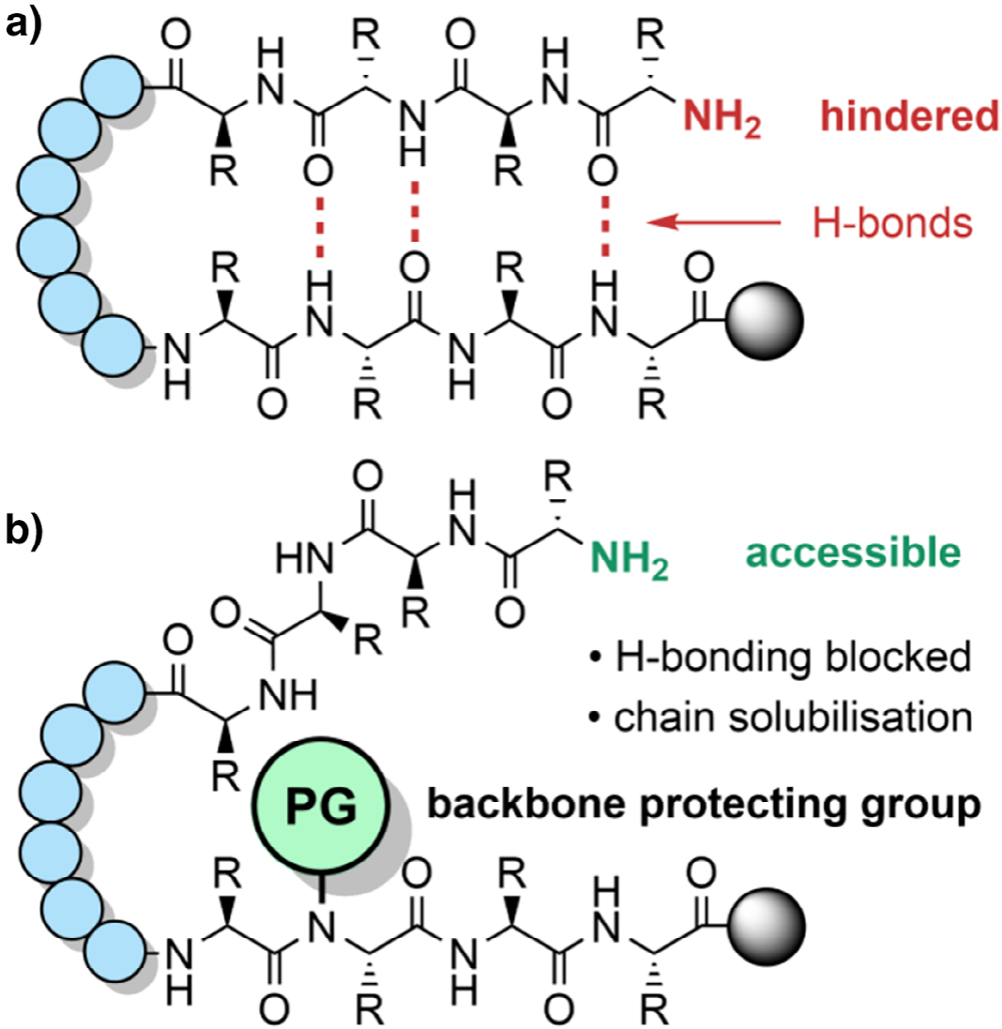
a) Peptide chain insolubility often leads to β‐sheet formation and a sterically hindered N‐terminus. b) The introduction of backbone protecting groups improves chain solubility and blocks β‐sheet formation, which ultimately improves peptide assembly.

While Fmoc SPPS may enable access to peptides >40 amino acids in length, the quality of the crude material is often drastically reduced, and the synthetic failure rate is much higher.^[^ ^55^ ^]^ Microwave‐assisted SPPS^[^ ^51^, ^56^ ^]^ and continuous flow methods^[^ ^57^, ^58^, ^59^ ^]^ have improved the synthesis of these longer sequences, although specialized equipment and large reagent excesses are required. The assembly of shorter peptides can also be inefficient, particularly for those that are rich in amino acids bearing aliphatic side chains.^[^ ^60^, ^61^ ^]^ These “difficult sequences” tend to solvate poorly on the solid support and aggregate, often by forming β‐sheets through hydrogen bonding of the peptide backbone.^[^ ^62^, ^63^, ^64^ ^]^ This leads to incomplete couplings and deprotections due to a sterically hindered N‐terminus, resulting in the formation of deletion sequences (Figure 1).^[^ ^32^, ^65^ ^]^ These peptidic impurities are often difficult to remove chromatographically due to their physicochemical similarities with the target peptide, which ultimately results in lower purities and yields.^[^ ^3^, ^55^ ^]^

**Scheme 2:**
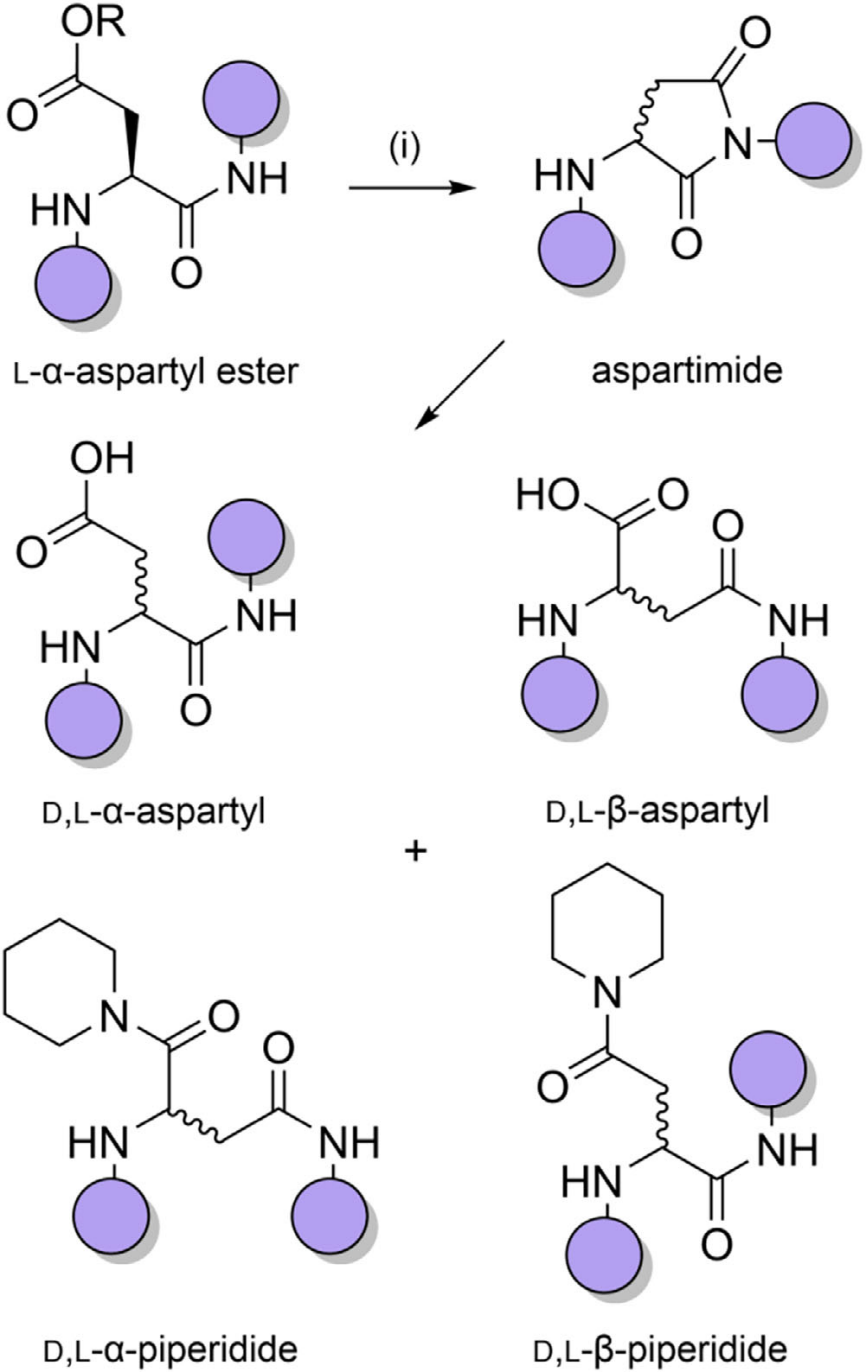
Base‐promoted aspartimide formation during Fmoc deprotection. i) 20% piperidine in DMF.

During *tert*‐butyloxycarbonyl (Boc) SPPS,^[^ ^66^ ^]^ this aggregation issue is not such a problem, as the TFA used for the Boc deprotections breaks up all previously formed secondary structures. Aggregation can reoccur when the protonated peptide is neutralized but can be ameliorated through in situ neutralization during coupling.^[^ ^67^ ^]^ However, there is no analogous strategy for Fmoc SPPS, which is now more widely used than Boc SPPS. Alternative approaches for suppressing chain aggregation include the use of chaotropic salts,^[^ ^68^ ^]^ highly polar solvent mixtures,^[^ ^69^, ^70^, ^71^ ^]^ and resins with functionalized linkers that improve solvation.^[^ ^72^, ^73^, ^74^, ^75^, ^76^ ^]^ A more effective strategy is to incorporate acid labile *N*‐protecting groups within the peptide backbone during chain assembly, to generate a tertiary amide bond. Backbone protection increases the solubility of the growing peptide chain in polar organic solvents such as DMF^[^ ^63^ ^]^ and disrupts β‐sheet H‐bonding,^[^ ^77^ ^]^ mimicking the effect of proline‐rich peptides (which are generally assembled efficiently).^[^ ^78^, ^79^, ^80^ ^]^ This approach is particularly effective when the protecting group is introduced at approximately every six residues.^[^ ^61^ ^]^ Backbone protection can also prevent base‐promoted aspartimide formation (Scheme 2),^[^ ^81^, ^82^, ^83^, ^84^ ^]^ enable epimerization‐free fragment condensations^[^ ^85^ ^]^ and promote peptide macrocyclizations (Table 1).^[^ ^86^ ^]^ TFA‐stable protecting groups with tunable acid lability are now used routinely to suppress peptide aggregation during purification^[^ ^87^ ^]^ and NCL reactions.^[^ ^88^ ^]^

**Table 1:** Benefits of backbone protection in Fmoc SPPS.

Application	Description
Peptide assembly	Enhanced yields are achieved through improved chain solubility and suppression of aggregation.
Macrocyclizations	Chain termini are proximal due to *cis* conformation of the protected amide, promoting cyclization. Backbone immobilization enables “on‐resin” macrocyclizations.
Preventing aspartimide	Formation of the succinimide intermediate is blocked.
Fragment condensations	Epimerization of the activated fragment can be suppressed. Solubility can also be enhanced.
C‐terminal modifications	Backbone immobilization enables the introduction of chemical diversity at the C‐terminus.
Solution‐phase handling	Aggregation can be suppressed during NCL and purifications, significantly improving yields.

Several peptide backbone protection strategies have been developed, each with their advantages and disadvantages. Benzyl‐based *N*‐protecting groups can theoretically be introduced into any dipeptide motif and thus can be used universally. However, these groups are often slow to cleave during TFA deprotections.^[^ ^89^ ^]^ Moreover, the reactive benzylic cations that are formed can alkylate sensitive residues on the deprotected peptide, such as cysteine or tryptophan.^[^ ^89^, ^90^ ^]^ Commercially available oxazolidine‐ and thiazolidine‐based pseudoproline dipeptides have also found widespread use, but are limited to serine, threonine, and cysteine‐rich peptides.^[^ ^91^, ^92^, ^93^ ^]^ The dicyclopropylmethyl (Dcpm) group is efficiently cleaved but its steric bulk limits its utility.^[^ ^94^ ^]^ The optimal backbone protecting group should be i) Fmoc SPPS compatible, ii) introduced efficiently, iii) sufficiently acid labile, and iv) universal in its applicability.

The scope of this review covers backbone protecting groups that are suitable for use in Fmoc SPPS. Key focus points will include their synthesis, incorporation, and effectiveness in improving peptide and protein assembly, suppressing side reactions, and enhancing macrocyclization yields. Protecting groups with tunable acid lability bearing solubilizing tags will also be examined in the context of solution‐phase handling and NCL. A commentary on the current state of the art will be provided, including the strengths and limitations of each backbone protecting group. Finally, future directions of the technology will be discussed in both industrial and academic research contexts.

## 2 Peptide Backbone Protection

### 2.1 Benzyl‐Based Protecting Groups

**Figure 2:**
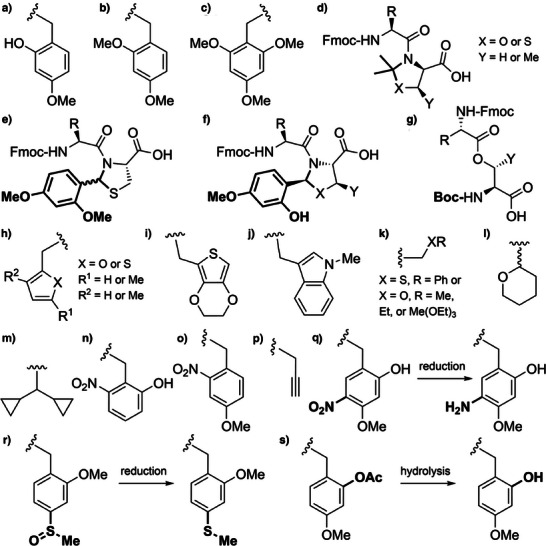
Backbone protecting groups for enhanced Fmoc SPPS. a) Hmb. b) Dmb. c) Tmb. d) Acetonide‐protected pseudoproline dipeptides. e) 2,4‐Dimethoxy‐*N*,*S*‐benzylidene‐protected cysteine pseudoproline dipeptides. f) *N*,*O*‐Benzylidene acetal‐protected dipeptides. g) Iso‐acyl dipeptides. h) Furfuryl and 2‐thienylmethyl. i) EDOTn. j) MIM. k) Alkoxymethyl and thiomethyl. l) Thp. m) Dcpm. n) 2‐Hydroxy‐6‐nitrobenzyl (2,6‐Hnb). o) 4‐Methoxy‐2‐nitrobenzyl. p) Prop. q) Hmnb can be reduced to 5‐amino‐2‐hydroxy‐4‐methoxybenzyl with CrCl_2_. r) Mmsb is reduced to the sulfide with NH_4_I. s) AcHmb is hydrolyzed to Hmb.

**Scheme 3:**
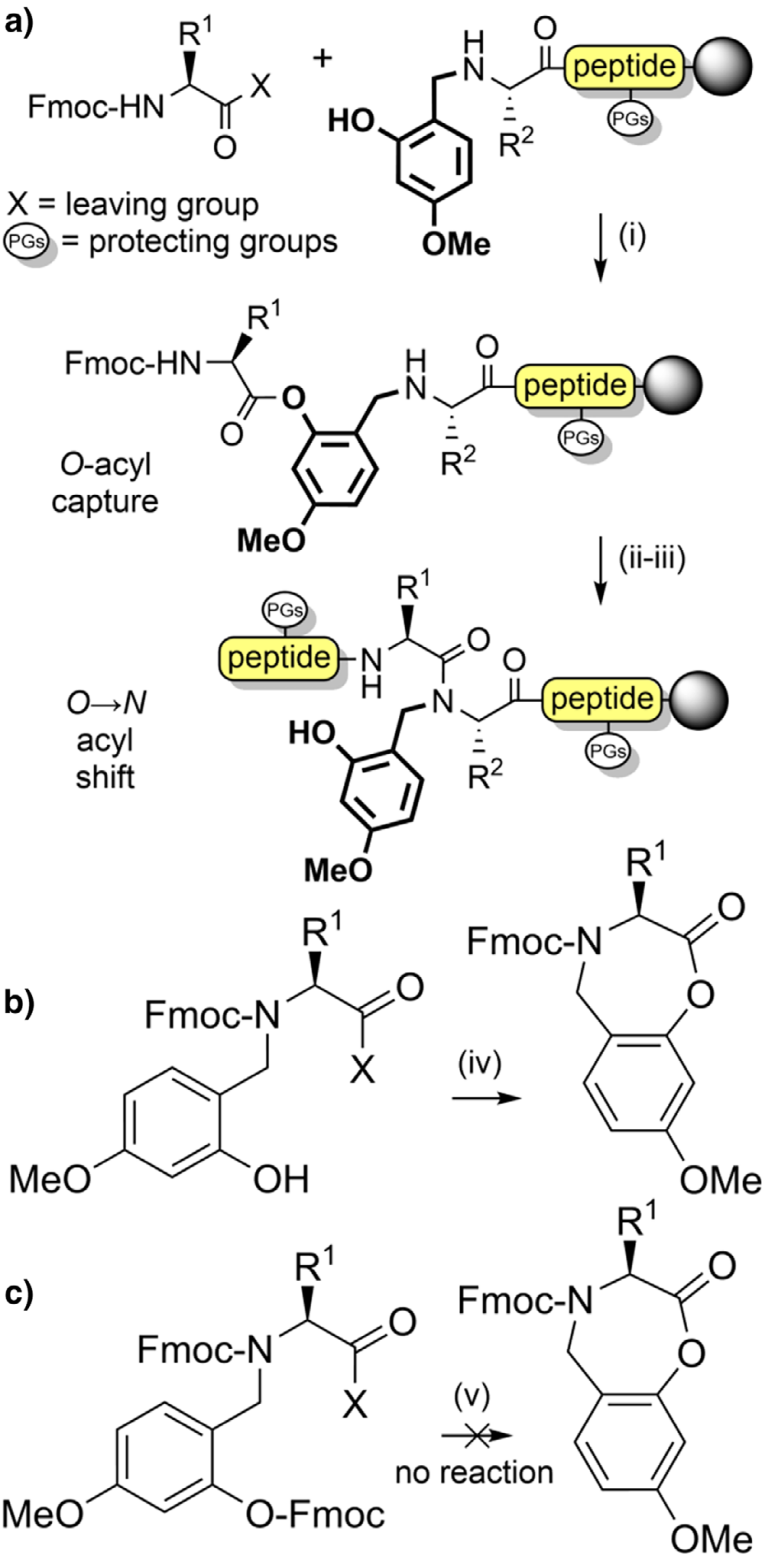
a) Acylation of a resin‐bound Hmb‐protected peptide N‐terminus. The backbone protecting group is depicted in bold font. i) *N*,*N*‐Diisopropylethylamine (DIEA). ii) DIEA. iii) Fmoc SPPS. b) Aryl lactonization side reaction during activation of Hmb‐protected Fmoc amino acids. iv) DIEA. c) *N*,*O*‐*Bis*‐Fmoc‐protected amino acids containing Hmb, to avoid lactonization. v) DIEA. X = leaving group.

The 2‐hydroxy‐4‐methoxybenzyl (Hmb) group (Figure 2a) is efficiently introduced into most *N* ^α^‐amides, although incorporation at junctions with flanking β‐branched side chains is challenging.^[^ ^95^ ^]^ Reductive amination of the corresponding benzaldehyde with the N‐terminal *N* ^α^‐amino group enables convenient in situ installation. The amino acid bearing the Hmb group can be effectively acylated by the incoming Fmoc‐amino acid, despite the sterically hindered secondary *N* ^α^‐amine. This is facilitated through acyl capture by the accessible 2‐hydroxyl group of Hmb, followed by an *O*→*N* acyl shift to generate the desired tertiary amide (Scheme 3a).^[^ ^89^, ^96^ ^]^ The Hmb group can also be introduced into peptides as *N*‐Fmoc Hmb amino acids,^[^ ^97^ ^]^ *N*,*O*‐*bis*‐Fmoc Hmb amino acids,^[^ ^89^ ^]^ their corresponding *O*‐pentafluorophenyl (OPfp) esters,^[^ ^90^, ^98^ ^]^ and through dipeptide building blocks.^[^ ^99^, ^100^ ^]^ To avoid formation of a cyclic aryl ester during activation of the *N*‐Fmoc Hmb amino acid, *O*‐protection of the Hmb phenol is generally preferable (Scheme 3b,c).^[^ ^97^ ^]^

**Scheme 4:**
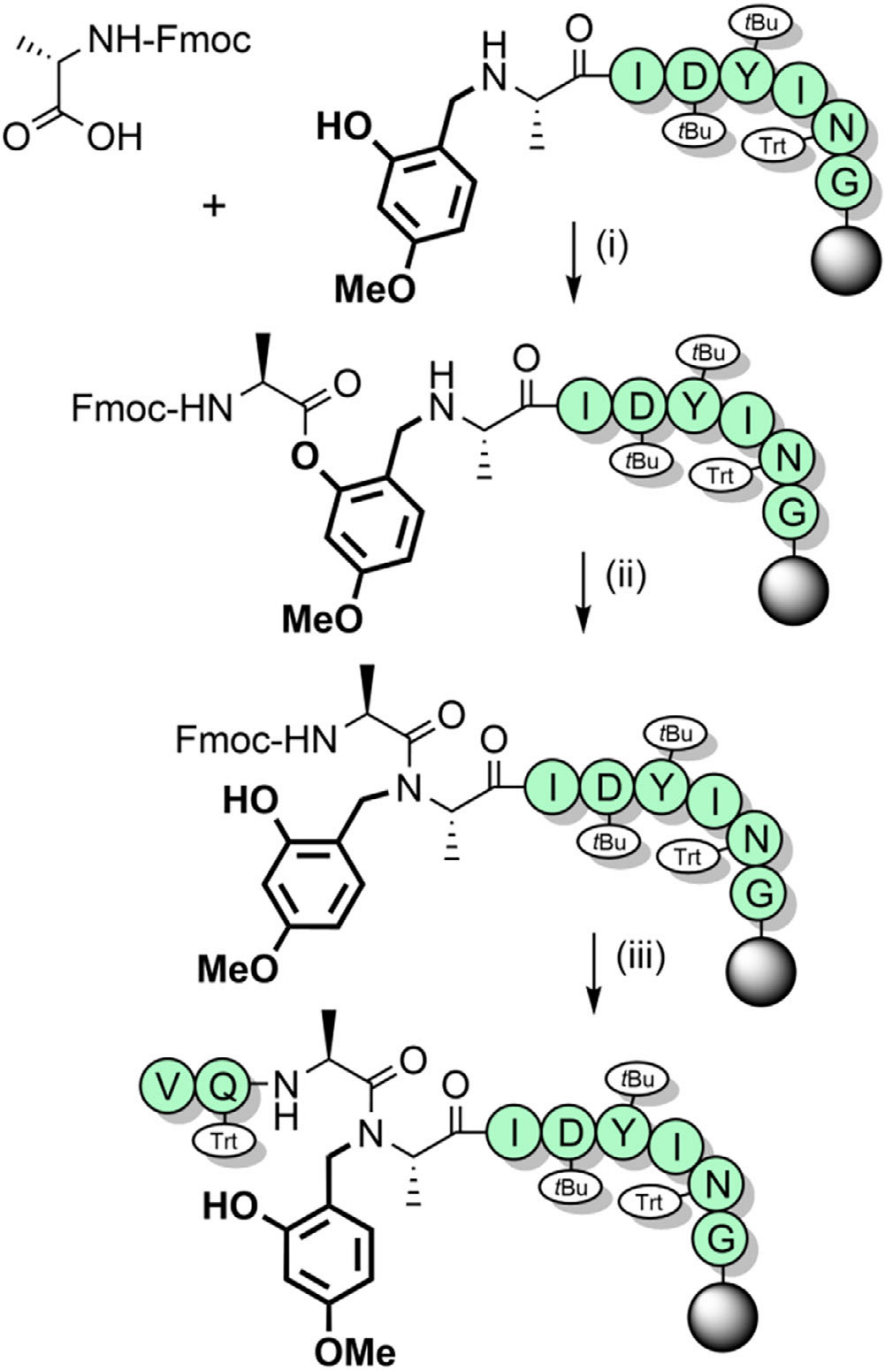
The synthesis of ACP(65–74) via Hmb protection. i) Fmoc‐Ala‐OH, ethyl‐*O‐*(1*H*‐benzotriazol‐1‐yl)uronium hexafluorophosphate, *O*‐(benzotriazol‐1‐yl)‐*N*,*N*,*N′*,*N′*‐tetramethyluronium hexafluorophosphate (HBTU), 1‐hydroxybenzotriazole (HOBt), *N*‐methylmorpholine (NMM), DMF. ii) O→N acyl shift. iii) Fmoc SPPS. *t*Bu = *tert*‐butyl, Trt = trityl.

Hmb backbone protection has improved the synthesis of numerous challenging peptides such as the acyl carrier protein fragment, ACP(65–74) (Scheme 4).^[^ ^61^, ^89^, ^96^, ^101^ ^]^ Using conventional Fmoc SPPS, the final valine addition is typically 10%–15% incomplete but with Hmb *N* ^α^‐protection at Ala^68^ the coupling proceeds to completion.^[^ ^89^, ^96^ ^]^ Fmoc‐(Hmb)Ala‐OH has been applied to the synthesis of both L‐ and D‐barnase (an RNA‐specific endonuclease), to investigate their chiral specificity. Hmb backbone protection is essential for efficiently obtaining peptide fragments of both barnase enantiomers, which were assembled via NCL.^[^ ^102^, ^103^ ^]^ Application of the Hmb group has also been shown to prevent aspartimide side products from forming, which commonly plagues synthesis of Asp–Asn^[^ ^104^ ^]^ and Asp–Gly^[^ ^81^, ^105^ ^]^ containing sequences.^[^ ^82^, ^106^ ^]^ Moreover, the Hmb group has also been applied to the synthesis of challenging purine‐rich peptide nucleic acids.^[^ ^107^ ^]^ The 2‐hydroxybenzyl group^[^ ^101^ ^]^ has also been utilized but is more acid stable than Hmb, requiring trifluoromethanesulfonic acid for its removal. This reduced acid lability is comparable to the 2‐mercaptobenzyl group^[^ ^108^ ^]^ which has been used as a thiol auxiliary for NCL.^[^ ^90^ ^]^ The TFA stability of these analogues deems them incompatible with conventional Fmoc SPPS methods; benzyl groups that are more electron‐rich are better suited.^[^ ^88^ ^]^

**Scheme 5:**
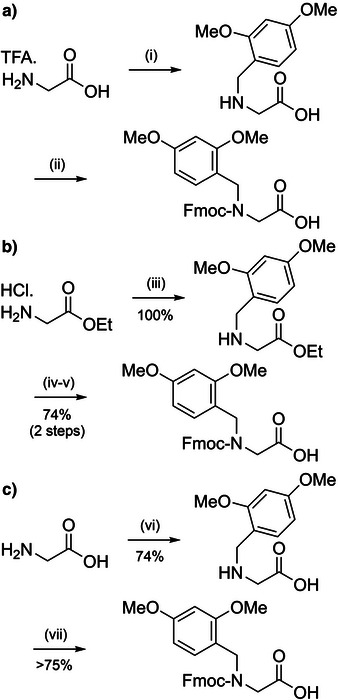
Various synthetic approaches for preparing Dmb‐protected dipeptides. a) i) 2,4‐Dimethoxybenzaldehyde, acetic acid, NaBH_3_CN. ii) Fmoc‐Cl, Na_2_CO_3_. b) iii) 2,4‐Dimethoxybenzaldehyde, triethylamine (TEA), NaBH(OAc)_3_. iv) 1 M NaOH. v) Fmoc succinate (Fmoc‐OSu), NaHCO_3_. c) vi) 2,4‐Dimethoxybenzaldehyde, KOH, NaBH_4_. vii) *N*,*O*‐*Bis*(trimethylsilyl)acetamide, Fmoc‐OSu, acidic workup.

**Figure 3:**
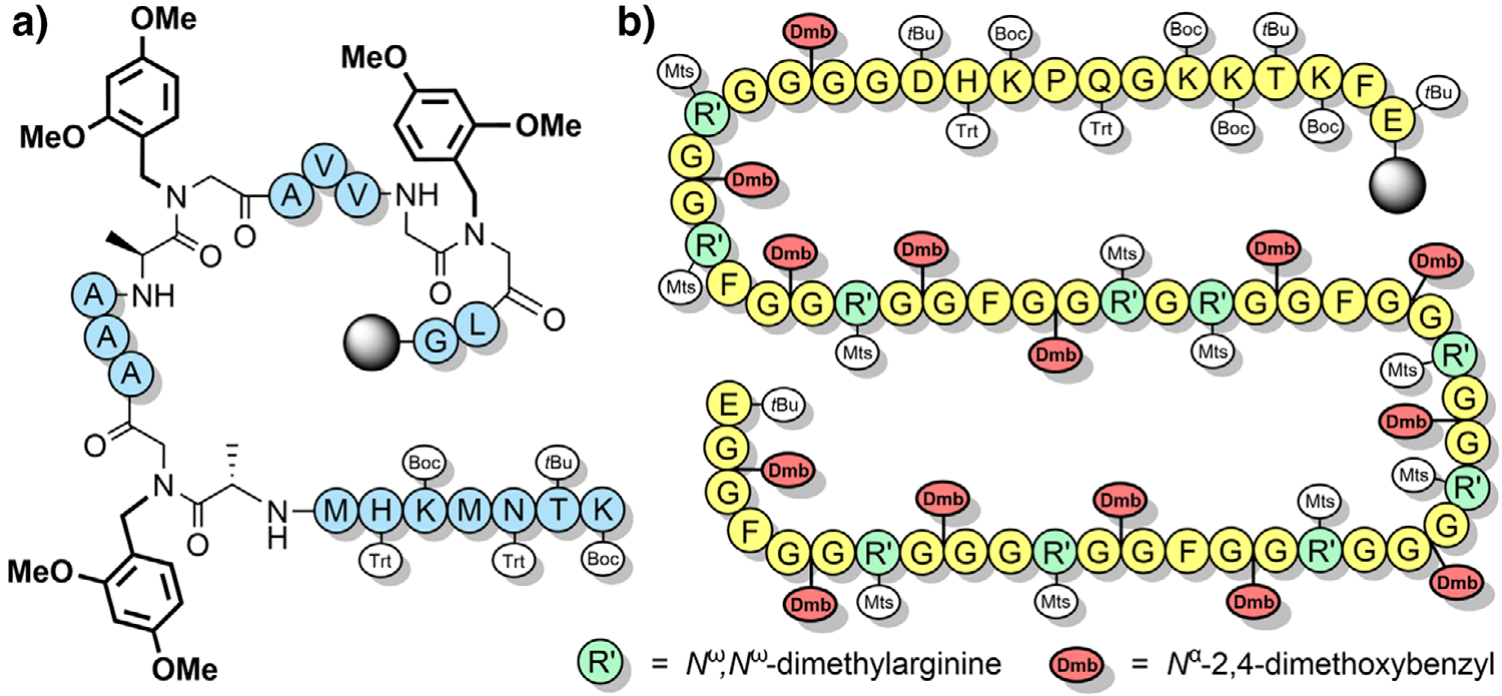
The application of Dmb backbone protection. a) PrP(106–126). b) C‐terminal region of human nucleolin. Mts = mesitylene‐2‐sulfonyl.

The 2,4‐dimethoxybenzyl (Dmb) group (Figure 2b) is also commonly used to improve peptide assembly^[^ ^84^ ^]^ and to prevent aspartimide formation^[^ ^83^ ^]^ for a range of “difficult” sequences.^[^ ^109^ ^]^ Dmb backbone protection has been shown to improve the solubility of protected peptide fragments in DMF and dichloromethane (DCM).^[^ ^80^ ^]^ Dmb has no reactive phenolic group that can compete with the N‐terminal amine during acylation, which is advantageous compared to Hmb. However, this also hampers efficient coupling (via *O*‐acyl capture) of sterically hindered amino acids, and therefore is largely limited to the *N*‐protection of glycine.^[^ ^110^ ^]^ Acylation of the Dmb‐protected N‐terminus can be improved through microwave heating,^[^ ^51^ ^]^ or by introducing Dmb‐containing dipeptides^[^ ^83^, ^111^ ^]^ during SPPS with the tertiary amide pre‐formed. The Dmb group is typically introduced during peptide assembly via commercially available Fmoc‐(Dmb)Gly‐OH,^[^ ^90^ ^],^ which can be efficiently prepared via a range of methods (Scheme 5).^[^ ^112^, ^113^ ^]^ Dmb dipeptides^[^ ^111^ ^]^ have been utilized to improve the assembly of hydrophobic peptides such as the neurotoxin prion fragment PrP(106–126) (Figure 3a),^[^ ^83^ ^]^ and the 61‐residue C‐terminal region of human nucleolin (Figure 3b). In the latter example, 14 Fmoc‐Gly‐(Dmb)Gly‐OH building blocks were used, which improved the high‐performance liquid chromatography (HPLC) yield from 5% to 26%.^[^ ^99^ ^]^ The Fmoc‐Asp(O*t*Bu)‐(Dmb)Gly‐OH dipeptide is also now widely used to prevent aspartimide formation.^[^ ^114^ ^]^ In one instance, crude purity was increased from 45% to 91% upon incorporation of this Dmb‐protected dipeptide unit.^[^ ^112^ ^]^

**Scheme 6:**
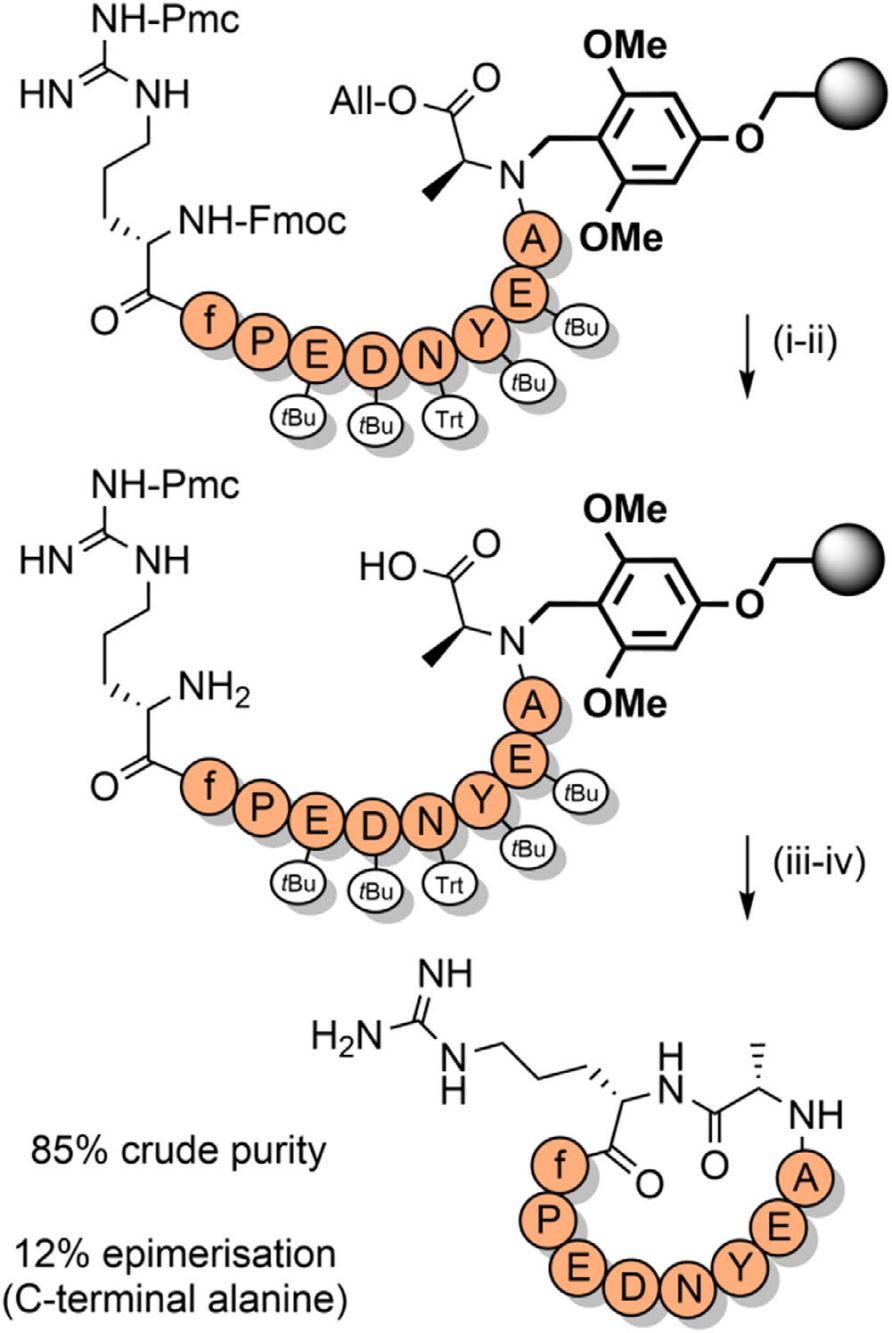
Cyclization on the solid support via a BAL. i) Pd(PPh_3_)_4_, morpholine, aqueous HCl, dimethylsulfoxide (DMSO), tetrahydrofuran (THF). ii) Piperidine, DMF. iii) Coupling reagents. iv) TFA cocktail. Pmc = 2,2,5,7,8‐pentamethylchroman‐6‐sulfonyl.

**Figure 4:**
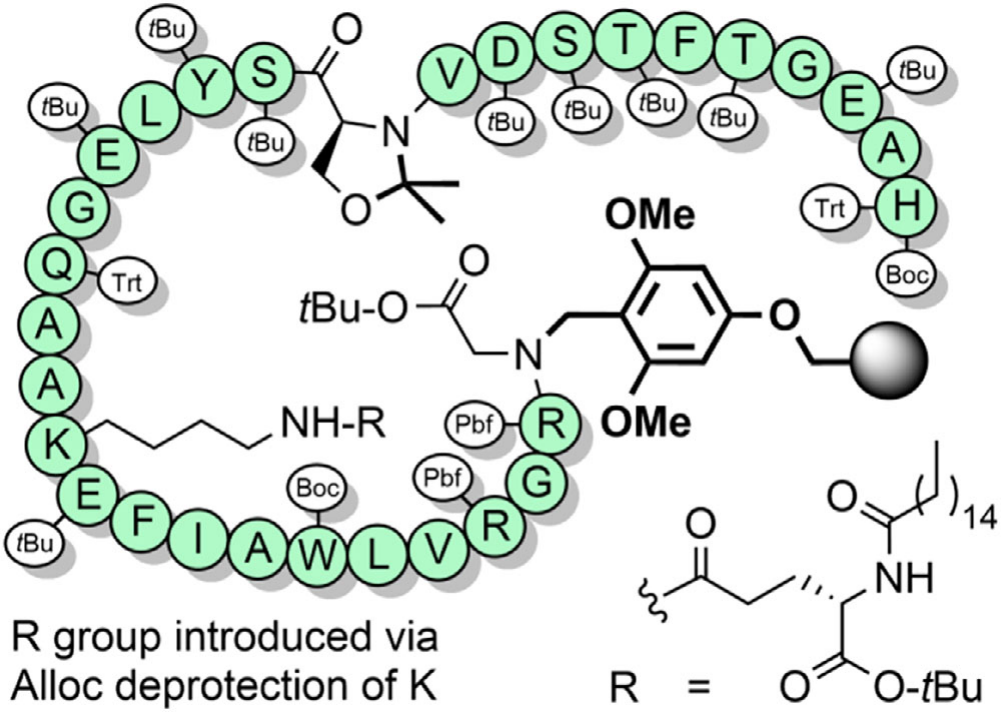
Scalable synthetic strategy for the preparation of liraglutide, using a BAL and a pseudoproline dipeptide. Pbf = 2,2,4,6,7‐pentamethyldihydrobenzofuran‐5‐sulfonyl.

The related 2,4,6‐trimethoxybenzyl (Tmb) group (Figure 2c) can be introduced to SPPS via Fmoc‐protected Ala or Gly precursors, which are prepared in up to 83% yield.^[^ ^90^, ^115^ ^]^ Tmb is more acid labile than Dmb because of the increased electron density of the ring, and also enhances coupling yields despite the additional steric bulk.^[^ ^90^ ^]^ This improvement is possibly due to an increase in the nucleophilicity of the *N* ^α^‐amino group, with acylation of Tmb‐protected glycine by the OPfp ester of Fmoc‐protected alanine being 90% complete after 1 h compared to 80% for Dmb‐protected glycine. An analogous trialkoxybenzyl motif has also been utilized as an acid‐labile backbone amide linker (BAL), whereby the C‐terminal residue is anchored through the *N* ^α^‐amino group.^[^ ^116^ ^]^ This allows for the introduction of chemically diverse C‐terminal groups such as aldehydes,^[^ ^117^ ^]^ and on‐resin macrocyclizations (Scheme 6).^[^ ^116^ ^]^ The BAL strategy has also been applied to the synthesis of liraglutide, to avoid side reactions and stability issues associated with the use of Wang and 2‐chlorotrityl linkers (Figure 4).^[^ ^116^, ^118^ ^]^ Isolated yields of up to 69% were obtained (which was also partly due to the use of pseudoproline dipeptides, see Section 2.2).

### 2.2 Pseudoprolines

**Scheme 7:**
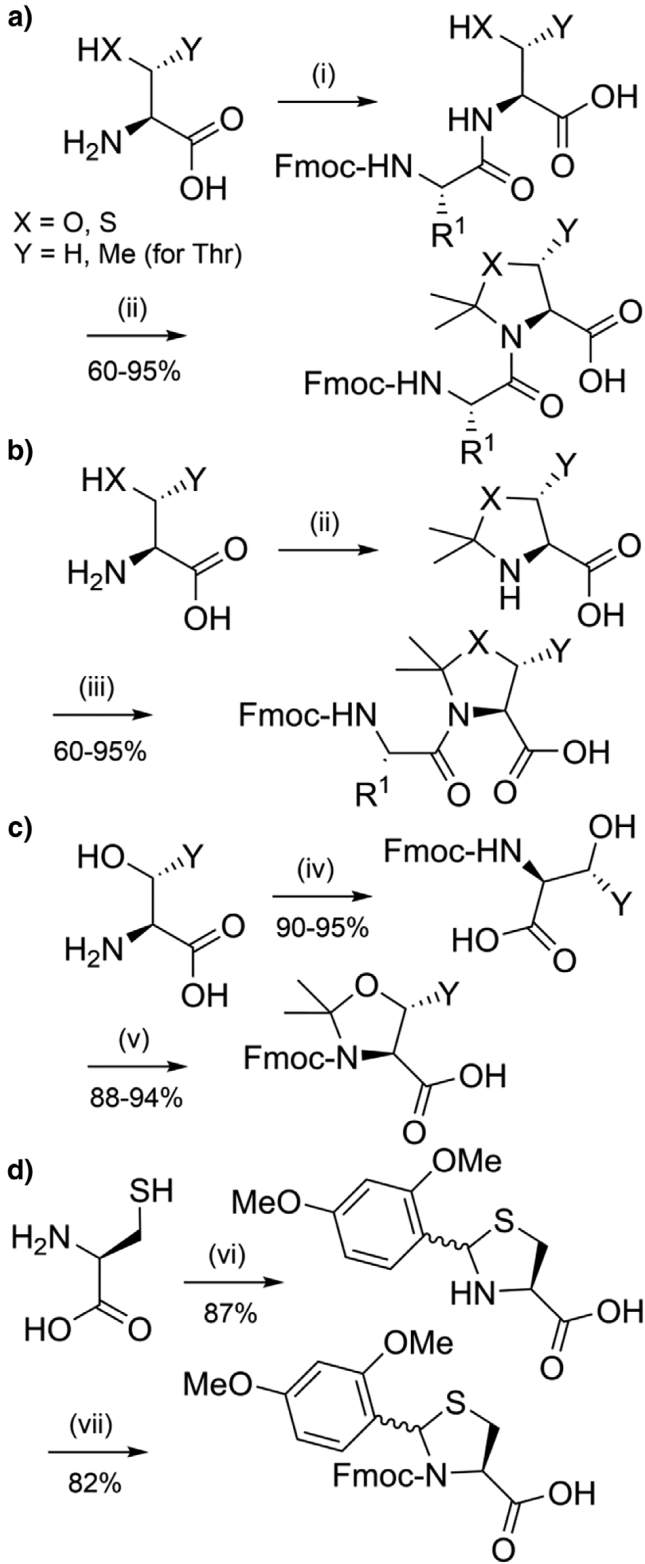
a) The synthesis of pseudoproline dipeptides. i) Fmoc amino acid OPfp ester, Na_2_CO_3_. ii) CH_2_O, Na_2_CO_3_, 2,2‐dimethoxypropane or acetone, pyridine *p*‐toluenesulfonate/BF_3_·Et_2_O/*p*TsOH. b) Method 2. iii) Fmoc amino acid fluoride or *N*‐carboxyanhydride, DIEA. c) Pseudoproline monomer synthesis. iv) Fmoc‐OSu, NaHCO_3_. v) 2,2‐dimethoxypropane, BF_3_·Et_2_O. d) Dmb‐protected Fmoc‐cysteine. vi) 2,4‐Dimethoxybenzaldehyde. vii) Fmoc‐OSu, Na_2_CO_3_.

Tertiary amide bonds formed by proline residues increase peptide solubility by reducing intra‐ and inter‐chain aggregation,^[^ ^78^, ^109^ ^]^ which served as inspiration for “pseudoprolines” (Figure 2d).^[^ ^91^, ^119^ ^]^ Commercially available Fmoc‐protected pseudoproline dipeptides consist of 5‐membered oxazolidine/thiazolidine rings that are synthesized through acid‐catalyzed reaction of serine, threonine or cysteine with 2,2‐dimethoxypropane (Scheme 7a,b).^[^ ^92^, ^120^, ^121^, ^122^ ^]^ The acetonide group acts as an acid‐labile protecting group for both the β‐hydroxyl/thiol group and the *N* ^α^‐amide. The two methyl substituents are essential for acid lability, with unsubstituted analogues being TFA‐stable.^[^ ^122^ ^]^ Pseudoprolines can also be efficiently introduced in SPPS as individual Fmoc‐amino acids (Scheme 7c). Subsequent coupling onto the hindered *N* ^α^‐amino group of the pseudoproline residue is possible, though double coupling^[^ ^93^ ^]^ or the utilization of flow chemistry^[^ ^123^ ^]^ is recommended. 2,4‐Dimethoxy‐*N*,*S*‐benzylidenes have also been introduced as pseudoproline‐like protection for cysteine (Figure 2e and Scheme 7d), and been applied to the preparation of therapeutically relevant peptides such as linaclotide.^[^ ^124^ ^]^ However, these Dmb‐protected cysteine‐based precursors must be introduced as dipeptides, to avoid inefficient coupling onto the sterically hindered *N* ^α^‐amino group.^[^ ^93^ ^]^

**Scheme 8:**
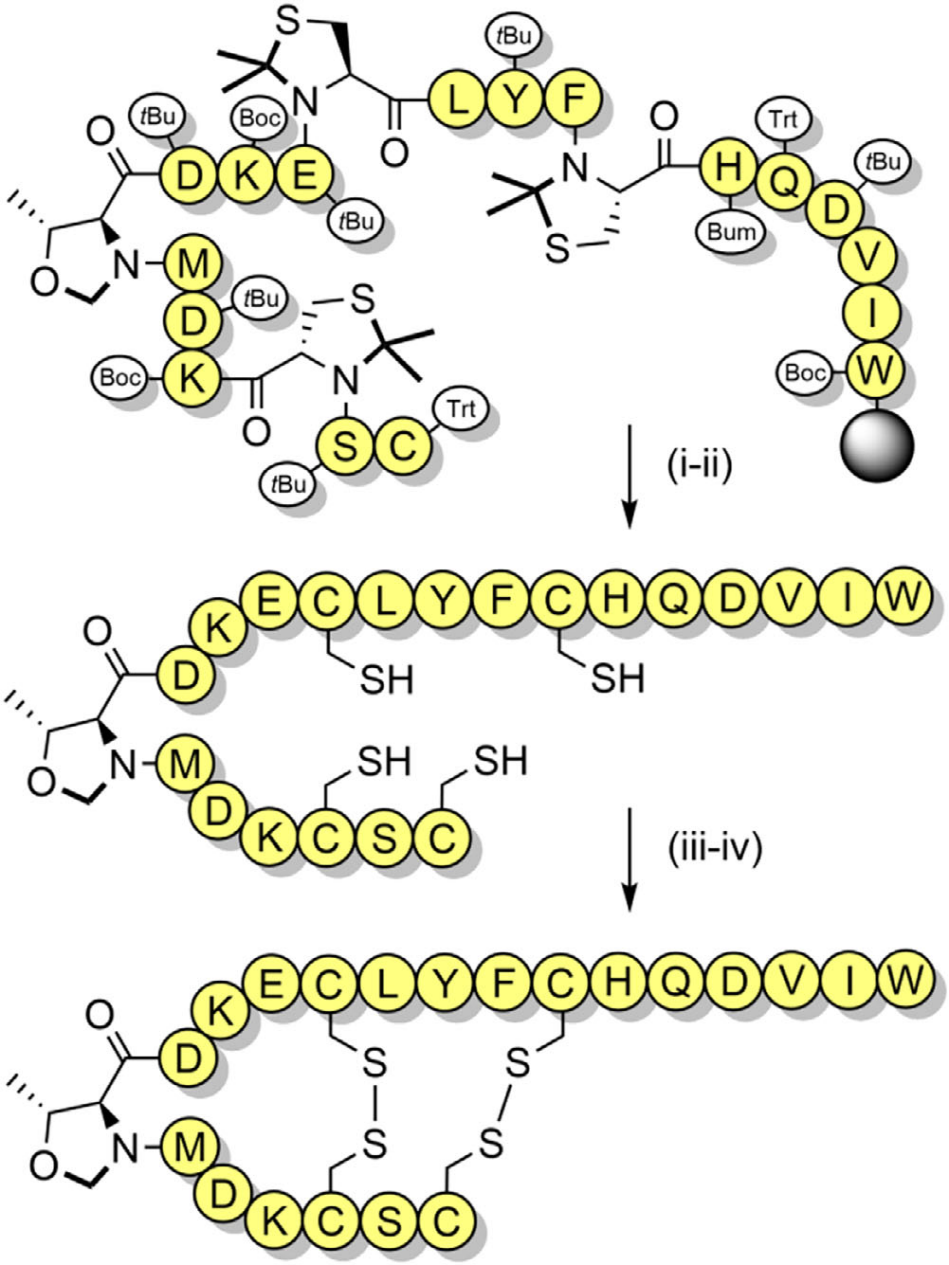
Synthesis and folding of sarafotoxin peptide using pseudoprolines. i) TFA (82.5%), 1,2‐ethanedithiol (2.5%), thioanisole (5%), H_2_O (5%), phenol (5%) for 2 h, then TFA (95%), H_2_O (5%) for 32 h. ii) Purification via RP‐HPLC. iii) Air oxidation, 3 h. iv) Purification via RP‐HPLC. Bum = 3‐[(1,1‐dimethylethoxy)methyl].

**Figure 5:**
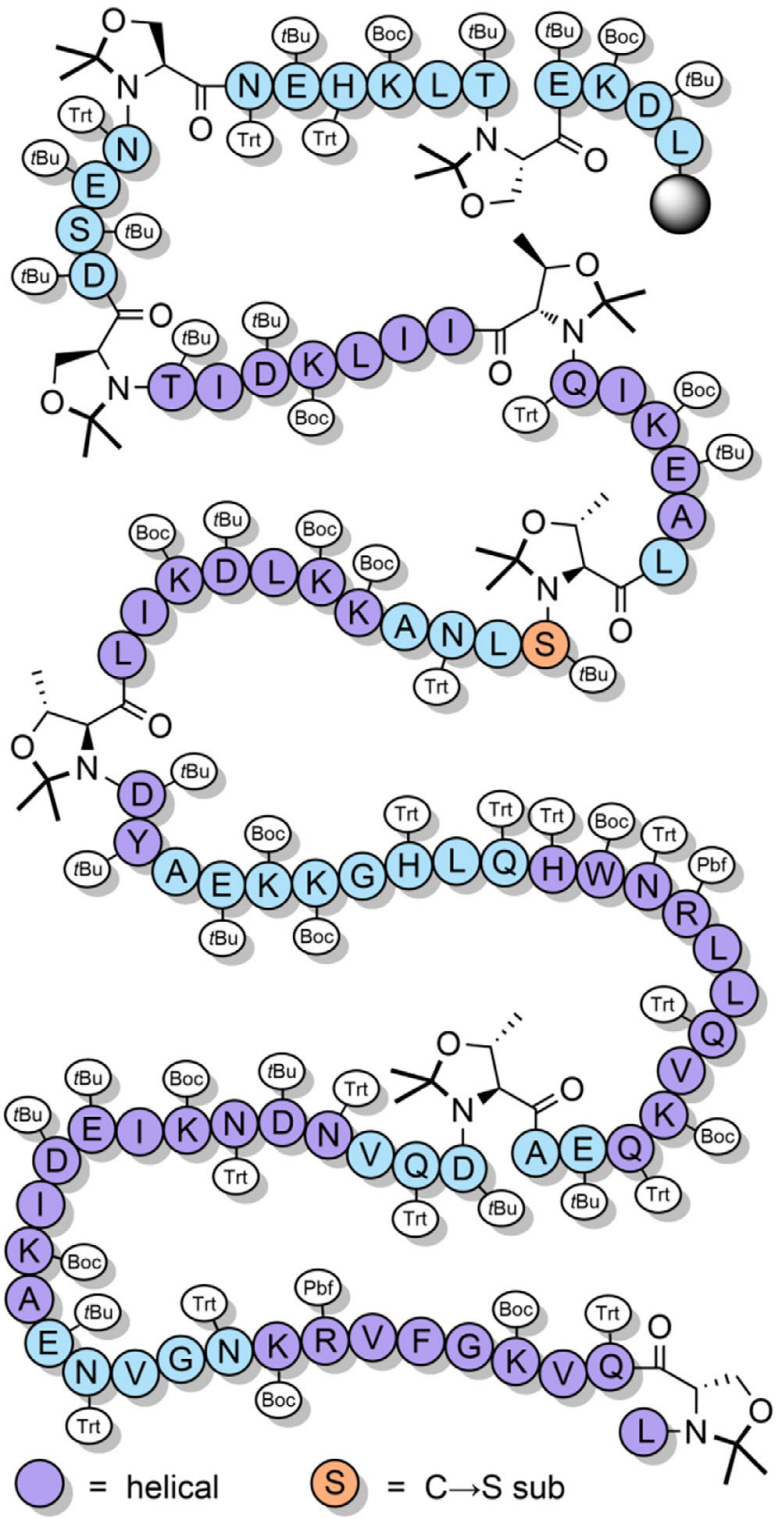
Utilization of pseudoprolines for the single‐shot synthesis of a 95‐residue FAS death domain protein fragment.

Serine, threonine, and cysteine pseudoprolines have been applied to a range of challenging peptide targets, such as macrocyclic peptides^[^ ^125^ ^]^ and short proteins.^[^ ^126^ ^]^ For example, a sarafotoxin analogue was assembled using three 2,2‐dimethylthiazolidine‐based precursors, with the 21‐residue linear peptide obtained in 18% yield.^[^ ^122^ ^]^ A TFA‐stable threonine‐based oxazolidine was also introduced, which induced a β‐turn and improved oxidative folding yields (Scheme 8). Pseudoprolines have also been applied to the single‐shot assembly of small proteins such as α‐synuclein(1–56), which is implicated in Parkinson's disease. Several isotopically labeled analogues were efficiently prepared for spectroscopic studies, by introducing three evenly spaced lysine‐threonine pseudoproline dipeptides.^[^ ^21^ ^]^ A range of serine and threonine pseudoproline dipeptides were also introduced during the synthesis of a 95‐residue FAS death domain protein fragment.^[^ ^127^ ^]^ The crude purity obtained was remarkable, given the large synthetic step count (Figure 5). Pseudoproline monomers have also been utilized to prepare human growth hormone, in 42% crude purity.^[^ ^128^ ^]^

Other notable works that have utilized pseudoproline dipeptides include the total chemical synthesis of a range of glycoproteins via NCL, such as erythropoietin (166 amino acids, 4 glycosylations),^[^ ^129^ ^]^ and the β‐subunits of *human* luteinizing hormone (121 amino acids, 1 glycosylation) and *human* chorionic gonadotropin (145 amino acids, 6 glycosylations).^[^ ^130^ ^]^ The introduction of *N*‐linked glycans can be particularly challenging as aspartyl protecting groups that are orthogonal to Fmoc SPPS, such as the *O*‐allyl group, are required. The allyl ester is sterically small and therefore particularly susceptible to nucleophilic attack by the backbone amide during base treatment, which leads to aspartimide. This can be avoided by introducing a pseudoproline vicinal to the aspartyl residue (on the C‐terminal side) if possible, to directly prevent succinimidyl formation.^[^ ^131^ ^]^ Alternatively, a pseudoproline may be introduced one residue earlier in the sequence, to induce a conformational change of the peptide backbone that is unfavorable for aspartimide formation.^[^ ^132^ ^]^ These examples highlight the effectiveness of the pseudoproline strategy for preparing long and difficult peptide and protein targets.

**Scheme 9:**
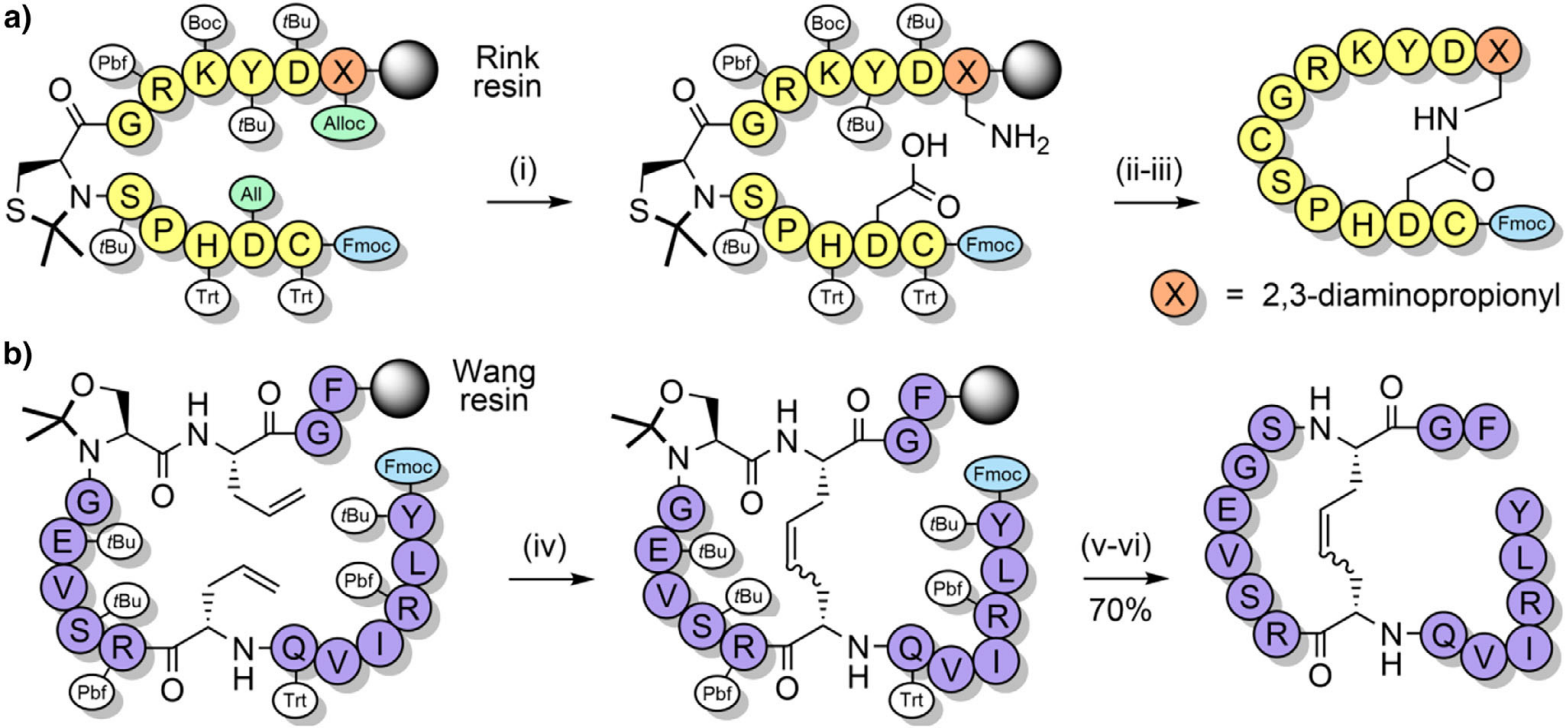
Synthesis of macrocyclic peptides via turn‐inducing pseudoproline moieties. a) Lactamization. i) PhSiH_3_, Pd(PPh_3_)_4_, N_2_. ii) Oxyma pure, diisopropylcarbodiimide (DIC), 2 h. iii) TFA. b) Ring‐closing metathesis. iv) Second generation Grubb's catalyst, CH_2_Cl_2_, LiCl in DMF, 66–72 h or microwave, 80 W (100 °C), 2 h. v) 20% piperidine/DMF, 20 m. vi) TFA (95%), H_2_O (2%), triisopropylsilane (TIPS, 2%), thioanisole (1%), 4 h. Alloc = allyloxycarbonyl. All = allyl.

**Scheme 10:**
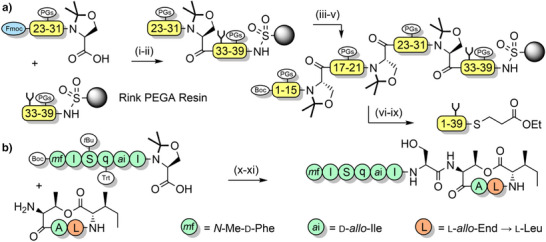
Epimerization‐free segment coupling. a) Synthesis of the N‐terminal domain of bovine ribonuclease C on the solid support. i) Benzotriazole‐1‐yl‐oxy‐tris‐pyrrolidino‐phosphonium hexafluorophosphate (PyBOP), DIEA, *N*‐methyl‐2‐pyrrolidone (NMP), microwave, 55 °C, 30 m. ii) 20% piperidine in NMP. iii) PyBOP, DIEA, NMP, microwave, 55 °C, 30 m. iv) 20% piperidine/NMP. v) PyBOP, DIEA, NMP, microwave. vi) (Trimethylsilyl)diazomethane, hexane/CH_2_Cl_2_. vii) Ethyl 3‐mercaptopropionate, PhSNa, DMF. viii) TFA, ethyl 3‐mercaptopropionate, Et_3_SiH, H_2_O. b) Solution‐phase fragment condensation to form Leu_10_‐teixobactin. ix) (1‐Cyano‐2‐ethoxy‐2‐oxoethylidenaminooxy)dimethylamino‐morpholino‐carbenium hexafluorophosphate (COMU), DIEA, dioxane, 60 °C, 2 h. x) TFA (97%), TIPS (1%), H_2_O (2%), 2 h.

**Scheme 11:**
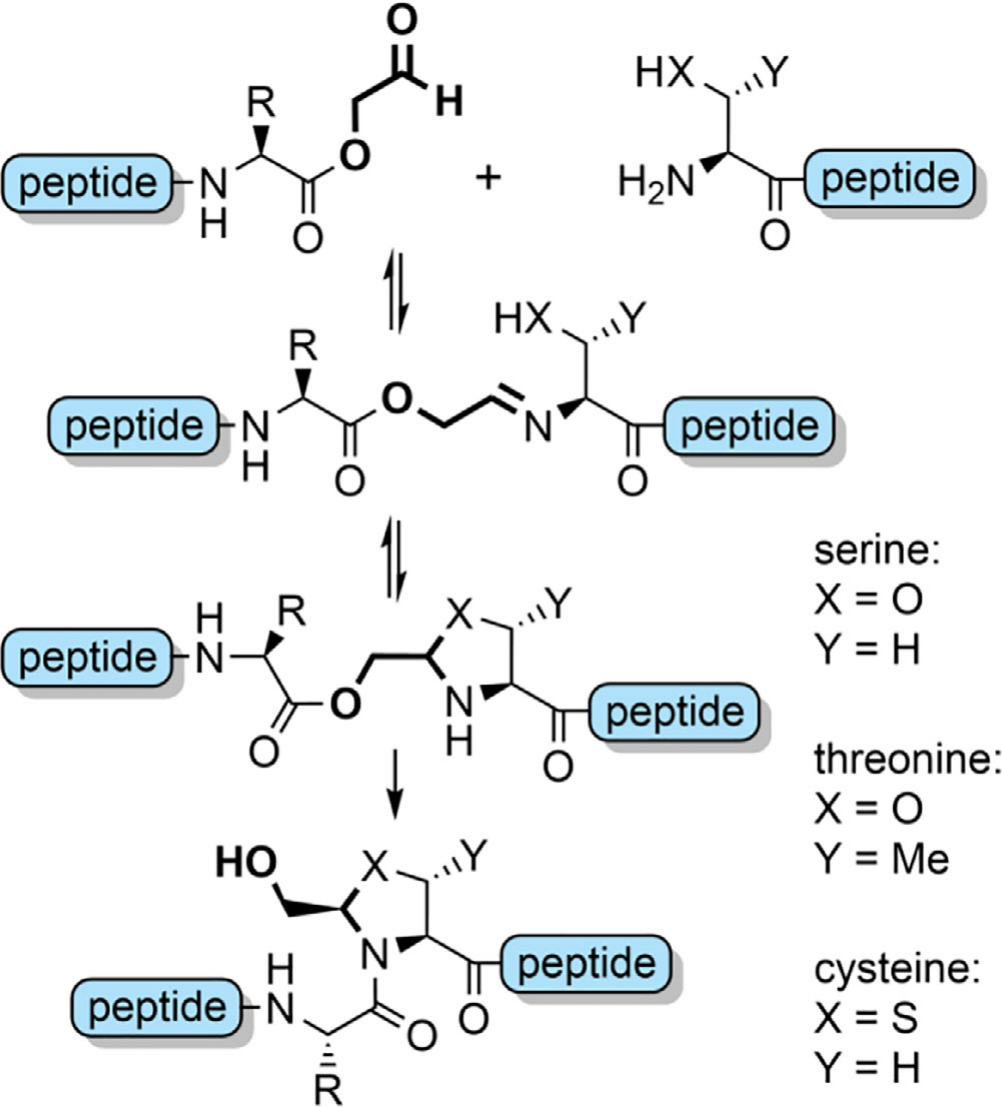
The ligation of a peptide bearing a C‐terminal glycoaldehyde ester with another containing an N‐terminal serine, threonine, or cysteine to form a pseudoproline junction.

Pseudoprolines in peptides favor a *cis*‐amide conformation,^[^ ^119^, ^121^, ^133^, ^134^ ^]^ with a *cis*/*trans* ratio of approximately 95:5.^[^ ^135^ ^]^ This is due to a steric clash between the substituent(s) at the C2‐position of the pseudoproline and the side chain of the N‐terminal vicinal residue, when in the *trans*‐amide conformation. The favoring of the *cis*‐amide conformation has a turn‐inducing effect on the peptide backbone, which can improve peptide macrocyclization yields by bringing the termini in close proximity to each other.^[^ ^136^, ^137^ ^]^ This approach has been applied to the synthesis of lactam peptides (Scheme 9a),^[^ ^86^ ^]^ and a dicarba analogue of a human growth hormone fragment which was cyclized via ring‐closing metathesis (Scheme 9b).^[^ ^138^ ^]^ Pseudoprolines have also been introduced at the C‐terminus of protected peptides to enable fragment couplings without epimerization. This strategy has been applied to the synthesis of the N‐terminal domain of bovine ribonuclease C on the solid support (Scheme 10a).^[^ ^139^ ^]^ Solution‐phase fragment condensations are also possible, such as in the case of the antimicrobial peptide teixobactin (Scheme 10b).^[^ ^85^ ^]^ Pseudoprolines can also be formed through the ligation of two unprotected peptide fragments: one bearing a C‐terminal glycoaldehyde ester, and another containing an N‐terminal serine, threonine, or cysteine (Scheme 11).^[^ ^140^ ^]^ Although the newly formed pseudoproline linkage is not native, it can be introduced as an isosteric replacement for proline, with minimal structural change.^[^ ^140^ ^]^ N‐terminal thiazolidines—which are TFA stable but are cleaved by nucleophiles such as methoxyamine—are also used as protection of cysteine during sequential NCL couplings of peptide fragments.^[^ ^141^, ^142^ ^]^ Despite pseudoprolines being limited to only three amino acids, they are versatile and extremely useful structural motifs in peptide and protein chemical synthesis.

**Scheme 12:**
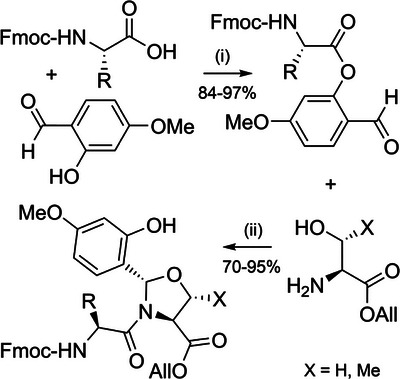
Synthesis of *N*,*O*‐benzylidene dipeptides. i) 1‐[*Bis*(dimethylamino)methylene]‐1*H*‐1,2,3‐triazolo[4,5‐] pyridinium 3‐oxide hexafluorophosphate (HATU), DIEA, DMF, 3 h. ii) Acetic acid/pyridine, CH_2_Cl_2_, 2–3 h.

**Figure 6:**
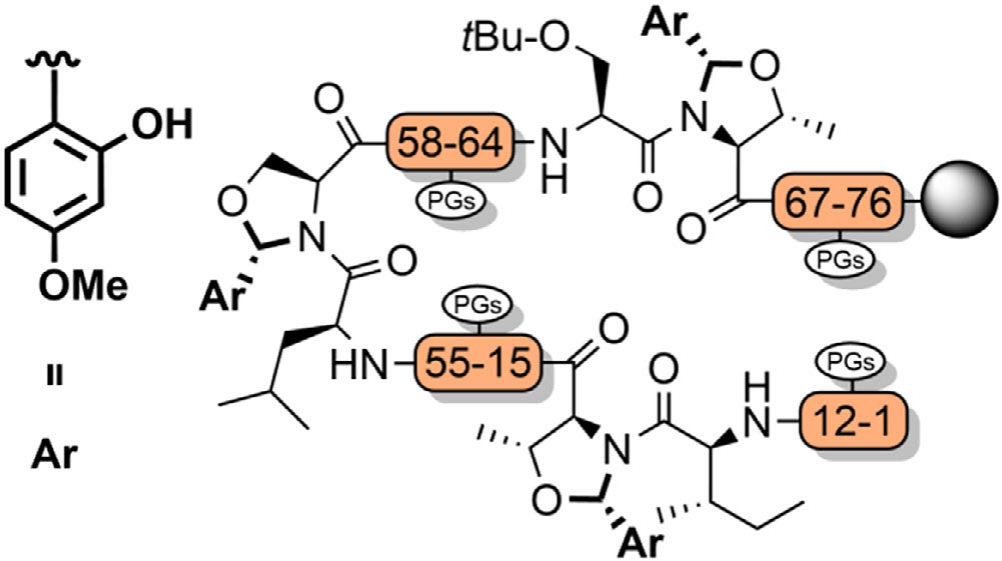
Synthetic strategy for the single‐shot synthesis of 76‐residue ubiquitin, via *N*,*O*‐benzylidene dipeptides.

Serine and threonine‐derived *N*,*O*‐benzylidene acetal dipeptides (Figure 2f) have a structure analogous to pseudoprolines, and elicit a similar effect in disrupting aggregation during peptide assembly.^[^ ^143^ ^]^ These building blocks are synthesized in excellent yield by coupling Fmoc amino acid 4‐methoxysalicylaldehyde esters with serine or threonine allyl esters (Scheme 12), via a reaction mechanism analogous to serine‐threonine protein ligations.^[^ ^29^ ^]^ To couple the dipeptide, allyl ester cleavage followed by activation of the resultant acid can be conducted in a one‐pot fashion. In situ incorporation on the solid support is also possible. The *N*,*O*‐benzylidene group is efficiently cleaved during global TFA deprotection, with the electron donating 4‐methoxy substituent increasing acid lability. *N*,*O*‐Benzylidene backbone protection has been applied to the single‐shot synthesis of a range of challenging peptides and small proteins, including the 76‐residue ubiquitin, which was obtained in 12% yield (Figure 6).^[^ ^143^ ^]^ In many cases, these dipeptides outperform pseudoprolines regarding crude peptide quality, ostensibly due to the kinked backbone.^[^ ^143^, ^144^ ^]^ *N*,*O*‐Benzylidene protection also assisted in the efficient preparation of four peptide fragments of histone H2B for NCL, in excellent yield. A similar strategy was applied to the assembly of erythropoietin.^[^ ^143^ ^]^ Notably, *N*,*O*‐benzylidene protection also suppressed aspartimide formation during Fmoc SPPS of the fragments, in addition to the improvements in peptide assembly.

### 2.3 Iso‐Acyl Dipeptides

**Scheme 13:**
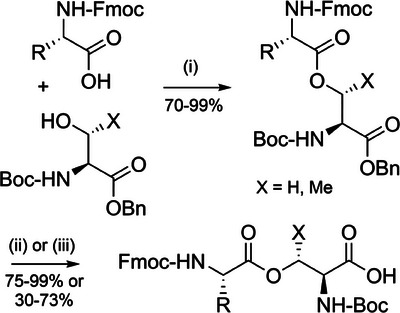
Synthesis of iso‐acyl Fmoc dipeptides. i) Fmoc amino acid, *N*‐ethyl *N*‐(3‐dimethylaminopropyl)‐carbodiimide hydrochloride (EDC·HCl), *N*,*N*‐dimethylaminopyridine (DMAP), CHCl_3_, 18 h. ii) Pd/C, H_2_, ethyl acetate, 18 h. iii) Pd/C, ammonium formate, EtOH–H_2_O (95:5), 40 °C, 3 h (for dipeptides containing methionine or cysteine).

Iso‐acyl linkages are ester bonds formed between the α‐carboxyl group of an amino acid and the side chain of a preceding *N* ^α^‐Boc‐protected serine or threonine residue.^[^ ^145^ ^]^ This non‐native, ester‐linked backbone disrupts chain aggregation during Fmoc SPPS and hence leads to significant improvement in the quality of crude peptides. Iso‐acyl groups can be introduced in situ on the solid support,^[^ ^146^ ^]^ or as protected dipeptide building blocks during Fmoc SPPS (Figure 2g).^[^ ^147^ ^]^ To avoid β‐elimination, a base‐free carbodiimide coupling in DCM is recommended.^[^ ^148^ ^]^ Diketopiperazine formation is also a risk, but this can be mitigated by using *N* ^α^‐amino protecting groups that can be cleaved with milder bases.^[^ ^149^, ^150^ ^]^ Iso‐acyl dipeptides are commercially available and use of these dipeptides is the preferred method for their incorporation, as the issue of epimerization during esterification is minimized. These dipeptides can be prepared in excellent yield in two steps (Scheme 13).^[^ ^147^ ^]^ Iso‐acyl linkages are TFA‐stable, which is advantageous as these depsipeptides are usually more soluble in acidic buffers. After purification, the iso‐acyl bond is transformed to the native peptide through an *O*→*N* acyl shift at pH 7.0 or above.^[^ ^151^ ^]^

**Scheme 14:**
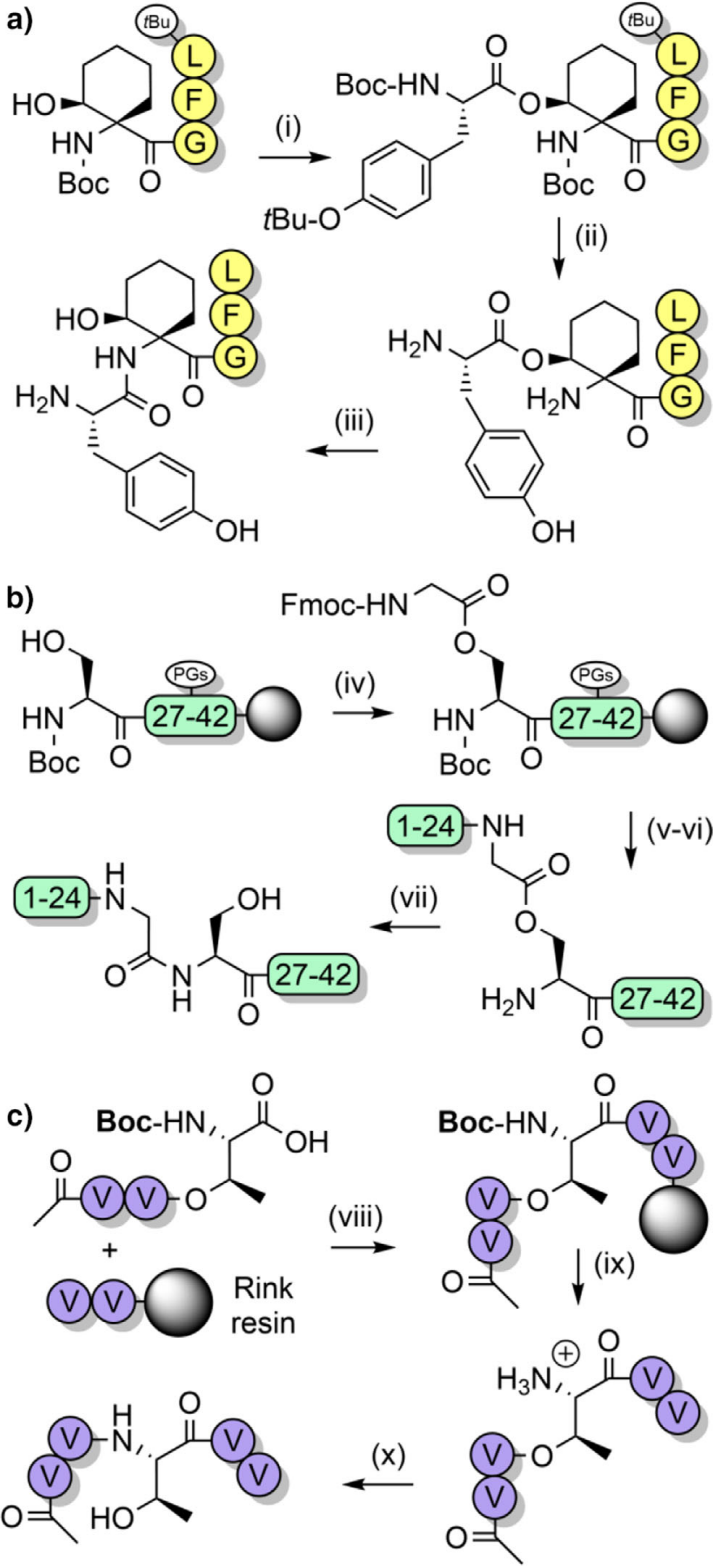
Application of the iso‐acyl method. a) Synthesis of α‐disubstituted enkephalin analogues. i) Boc‐Tyr(*t*Bu)‐OH, EDC·HCl, DMAP, CH_2_Cl_2_, 2 h. ii) TFA, CH_2_Cl_2_, 0 °C, 1 h. iii) 10% NH_4_HCO_3(aq)_. b) Synthesis of Aβ(1–42). iv) Fmoc‐Gly‐OH, DIC, DMAP, CH_2_C1_2_, 4 h × 2. v) Fmoc SPPS. vi) TFA (92.5%), *m*‐cresol (2.5%), thioanisole (2.5%), H_2_O (2.5%). vi) pH 7.4. c) Epimerization‐free fragment condensation. viii) DIC, HOBt, DMF. ix) TFA (92.5%), *m*‐cresol (2.5%), thioanisole (2.5%), H_2_O (2.5%), 1.5 h. x) Phosphate buffered saline, pH 7.4, 25 °C.

The iso‐acyl method has been applied to a range of challenging peptide targets to improve purity.^[^ ^147^, ^150^, ^152^ ^]^ For example, Leu‐enkephalin analogues bearing hindered α,α‐disubstituted serine variants at position 2 were efficiently prepared via initial coupling of the N‐terminal tyrosine through an iso‐acyl ester linkage to the β‐hydroxyl group of the serine analogue. The target peptides were obtained after global deprotection, followed by *O*→*N* acyl shifts (Scheme 14a).^[^ ^153^ ^]^ Iso‐acyl linkages have also greatly improved the preparation of the highly insoluble and aggregation‐prone Aβ(1–42) and its analogues, which is implicated in Alzheimer's disease.^[^ ^146^, ^149^, ^154^, ^155^ ^]^ By substituting the Gly^25^–Ser^26^ moiety for an iso‐acyl linkage, the peptide's water solubility is increased substantially compared to the native peptide,^[^ ^154^ ^]^ with long‐term storage possible.^[^ ^156^, ^157^ ^]^ Synthetic iso‐acyl Aβ(1–42) can be prepared in high purity, with conversion to the fibrilization‐prone Aβ peptide initiated through the final‐stage *O*→*N* acyl shift (Scheme 14b). Iso‐acyl dipeptides have also been utilized in the synthesis of insulin, which readily aggregates in solution (A‐chain precursors are particularly problematic).^[^ ^158^ ^]^ For chemical assembly, numerous synthetic steps are required to form each of insulin's disulfide bonds sequentially. This results in extremely poor yields, due to aggregation during this excessive handling. By introducing iso‐acyl dipeptides into both the A‐chain and the B‐chain, synthetic yields were increased from 15% to 68%.^[^ ^159^ ^]^ Their use in the A‐chain is essential, particularly for precursors bearing hydrophobic *S*‐protecting groups such as 2‐nitroveratryl.^[^ ^160^, ^161^ ^]^

Iso‐acyl dipeptides have been incorporated at the C‐terminus of protected peptide fragments for epimerization‐free fragment couplings (Scheme 14c).^[^ ^148^, ^151^, ^162^ ^]^ Epimerization is reduced in this process as the C‐terminal amino acid is urethane‐protected, which destabilizes the intermediates that lead to epimerization.^[^ ^163^ ^]^ However, the iso‐acyl dipeptides are prone to β‐elimination in the presence of organic bases; therefore, carefully controlled conditions for Fmoc removal are advisable.^[^ ^148^ ^]^ Although the iso‐acyl method is limited to serine and threonine residues, they are a useful alternative to other backbone protecting groups, especially for peptides that are aggregation‐prone in solution.

### 2.4 Heterocyclic Protecting Groups

**Scheme 15:**
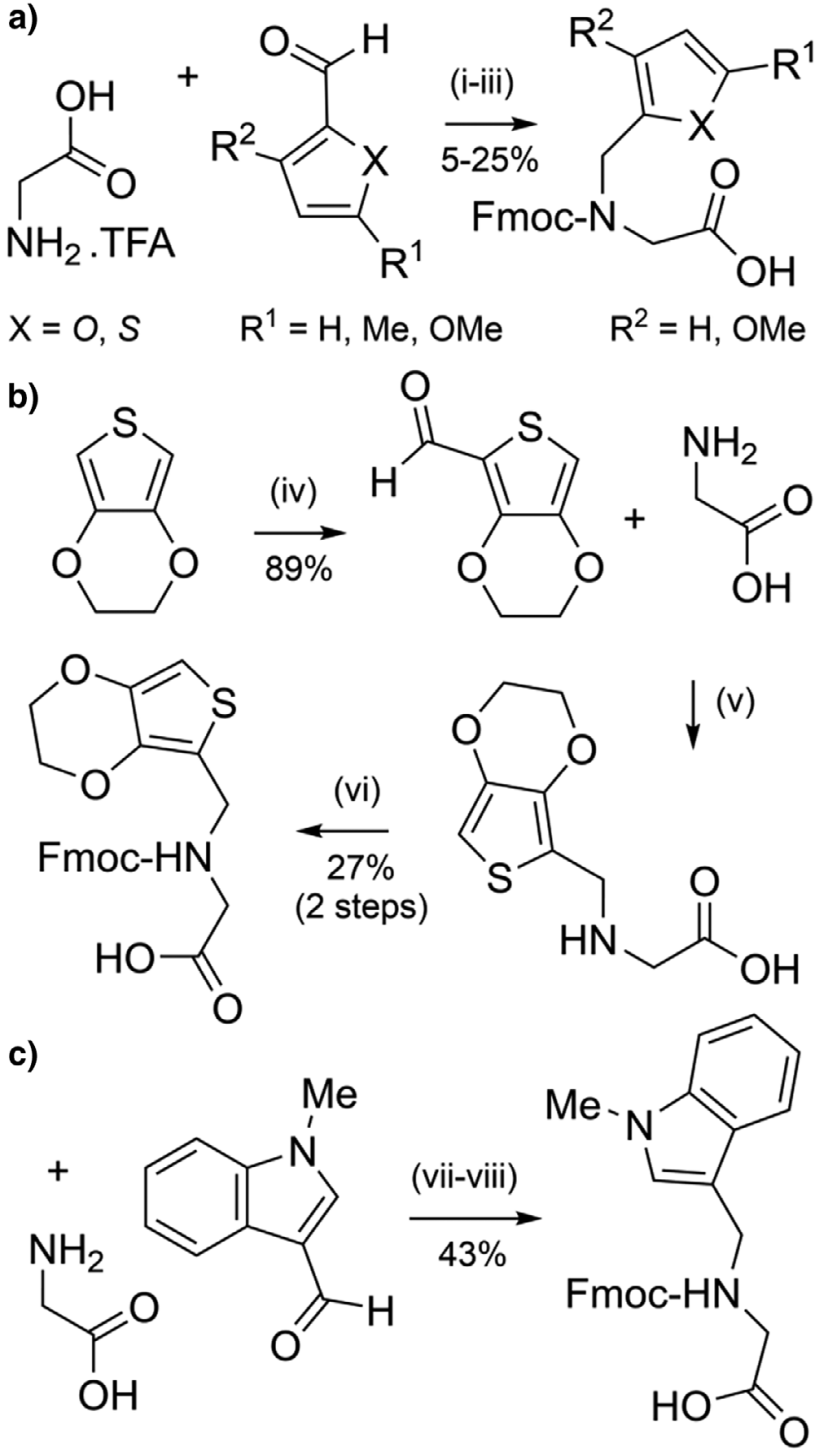
Synthesis of heterocyclic‐based backbone‐protected Fmoc amino acids. a) Furfuryl and 2‐thienylmethyl protection. i) Acetic acid/methanol. ii) NaBH_3_CN. iii) Na_2_CO_3_, Fmoc‐Cl. H_2_O/dioxane. b) EDOTn protection. iv) *n*‐BuLi, −78 °C, DMF, THF, Ar. v) NaBH_3_CN, H_2_O/dioxane, pH 5–6. vi) Fmoc‐Cl, Na_2_CO_3_, H_2_O/dioxane, pH 8–10. c) MIM protection. vii) NaBH_3_CN, H_2_O/dioxane, pH 5–6. viii) Fmoc‐Cl, Na_2_CO_3_, H_2_O/dioxane, pH 8–10.

A range of heteraromatic‐based *N*‐protecting groups, analogous to *N*‐benzyl type groups, have been investigated for both improved yields of incorporation and their cleavage kinetics. Several substituted furfuryl‐ and 2‐thienylmethyl‐based protecting groups have been evaluated (Figure 2h) and benchmarked against Dmb and Tmb.^[^ ^90^ ^]^ The rationale for this study was to develop smaller protecting groups with improved coupling efficiency. The protected amino acids were prepared in modest yields via reductive amination followed by Fmoc protection (Scheme 15a). The most acid labile protecting group was 5‐methoxythienylmethyl, which was comparable to Tmb. The 5‐methoxyfurfuryl group was unstable in TFA and not investigated further. Unfortunately, acylation of all furfuryl and 2‐thienylmethyl‐protected α‐amino groups on the solid support was significantly less successful than with the benzyl‐based groups. For example, only 25% of the 5‐methoxythienylmethyl‐protected amine was acylated, compared with 80% and 90% for the Dmb and Tmb‐protected amines, respectively. Both furfuryl‐ and 2‐thienylmethyl‐based protecting groups require further optimization and are likely to be limited to *N* ^α^‐protection of glycine and alanine.

3,4‐Ethylenedioxy‐2‐thienyl (EDOTn) and 1‐methyl‐3‐indolylmethyl (MIM) have also been investigated as backbone protecting groups (Figure 2i,j).^[^ ^164^ ^]^ Both can be incorporated onto glycine via reductive amination from the corresponding aldehyde precursors, with the Fmoc‐protected glycine derivatives obtained in 24% (three steps) and 43% (three steps) yield, respectively (Scheme 15b,c). Both EDOTn and MIM are more acid labile than Dmb, and effective in preventing aspartimide formation. Moreover, coupling onto the EDOTn‐protected α‐amine was more efficient than coupling onto the Dmb‐protected α‐amine, possibly due to an increase in the nucleophilicity of the amino group. However, coupling efficiency onto the MIM‐protected α‐amine was inefficient, which is most likely caused by the bulky *N*‐methylated indole, suggesting that EDOTn is more viable as an Fmoc SPPS compatible backbone protecting group.^[^ ^164^ ^]^

### 2.5 *N*,*O‐* and *N*,*S*‐Acetal Protecting Groups

**Scheme 16:**
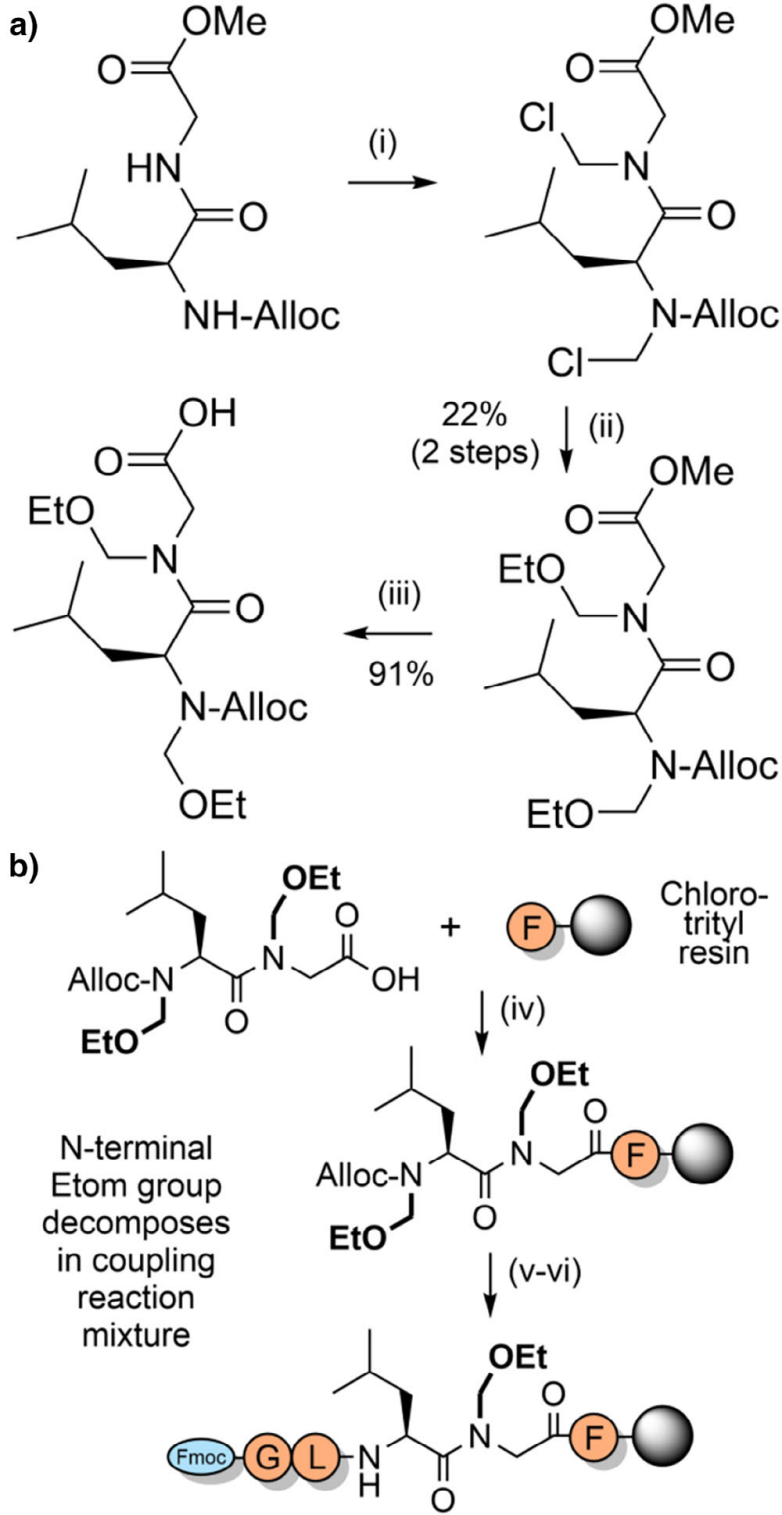
Alkoxymethyl backbone protection. a) Synthesis of Etom‐protected Alloc dipeptides. i) (CH_2_O)*_n_*, SOCl_2_, 18 h. ii) NaHCO_3_, DMAP, ethanol, 18 h. iii) LiOH, H_2_O/dioxane (2:3), 30 m. b) Fmoc SPPS using Etom‐protected dipeptides. iv) DIC, Oxyma pure, DMF, 1 h. v) Pd(PPh_3_)_4_, PhSiH_3_, CH_2_Cl_2_. vi) Fmoc SPPS.

*N*‐Alkoxymethyl and *N*‐alkylthiomethyl backbone protecting groups are highly acid labile and can be prepared by a Mannich reaction between formaldehyde and an alcohol or thiol directly onto the Fmoc‐amino acid.^[^ ^165^ ^]^ Some examples include phenylthiomethyl (Ptm), methoxymethyl (Mom), and triethylene glycol monomethyl ether (Tegom) (Figure 2k). Tegom contains an oligoethylene glycol chain which serves as an additional aggregation‐disrupting moiety.^[^ ^166^ ^]^ The Ptm group was applied to the synthesis of the challenging Ala_13_ oligomer, which was prepared in excellent yield.^[^ ^165^ ^]^ *N*‐Alkoxymethyl and *N*‐alkylthiomethyl‐protected amino acids and dipeptides can also be accessed by electrochemical oxidation of *N*‐silylmethyl,^[^ ^167^ ^]^ and through nucleophilic attack of *N*‐chloromethyl intermediates.^[^ ^168^ ^]^ The latter method—whereby the *N*‐chloromethyl group is incorporated via reaction with thionyl chloride and formaldehyde—is particularly effective for preparing *N*‐alkoxymethyl‐protected Alloc amino acids (Scheme 16a). However, this method is limited to aliphatic amino acids such as glycine, alanine, and leucine, due to the harsh reactions conditions required. Coupling onto the resin‐bound *N*‐alkoxymethyl amine also proved to be challenging due to loss of the alkoxymethyl group; the acyl chloride method was modestly successful but only approximately 20% of the protecting group remained. Efficient acylation was eventually achieved via a dipeptide strategy, through *bis*‐*N*‐ethoxymethyl (Etom) protection (Scheme 16b). *N*‐Alkoxymethyl groups were found to be highly acid labile and thus they have significant potential as backbone protecting groups in Fmoc SPPS with further optimization.

**Scheme 17:**
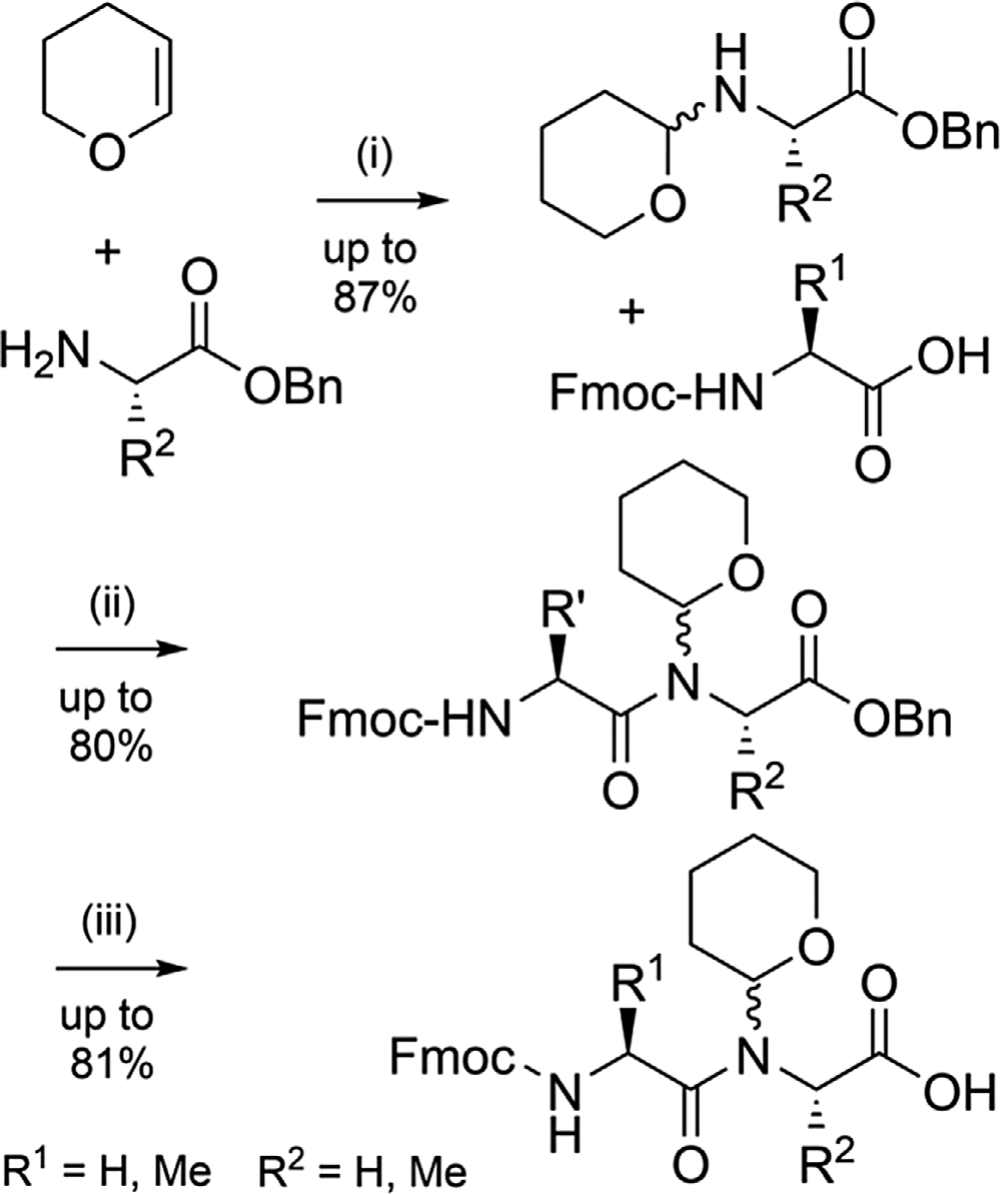
Synthesis of Thp‐protected Fmoc dipeptides. i) 1 N HCl_(aq)_, 1 h. ii) Fmoc amino acid activation: 2.2 equiv Fmoc‐Gly‐OH, 2.2 equiv NMM, 2.2 equiv isobutylchloroformate (IBCF) in DMF, 0 °C for 20 m. Dipeptide coupling: 1 equiv Thp‐protected amino benzyl ester, 1 equiv *N*,*O*‐*bis*(trimethylsilyl)acetamide in DMF (pre‐activated for 20 m), then added to the activated Fmoc amino acid. iii) 10% w/w Pd(OH)_2_/C, H_2_ (5–15 bar), methanol, 5–24 h.

**Figure 7:**
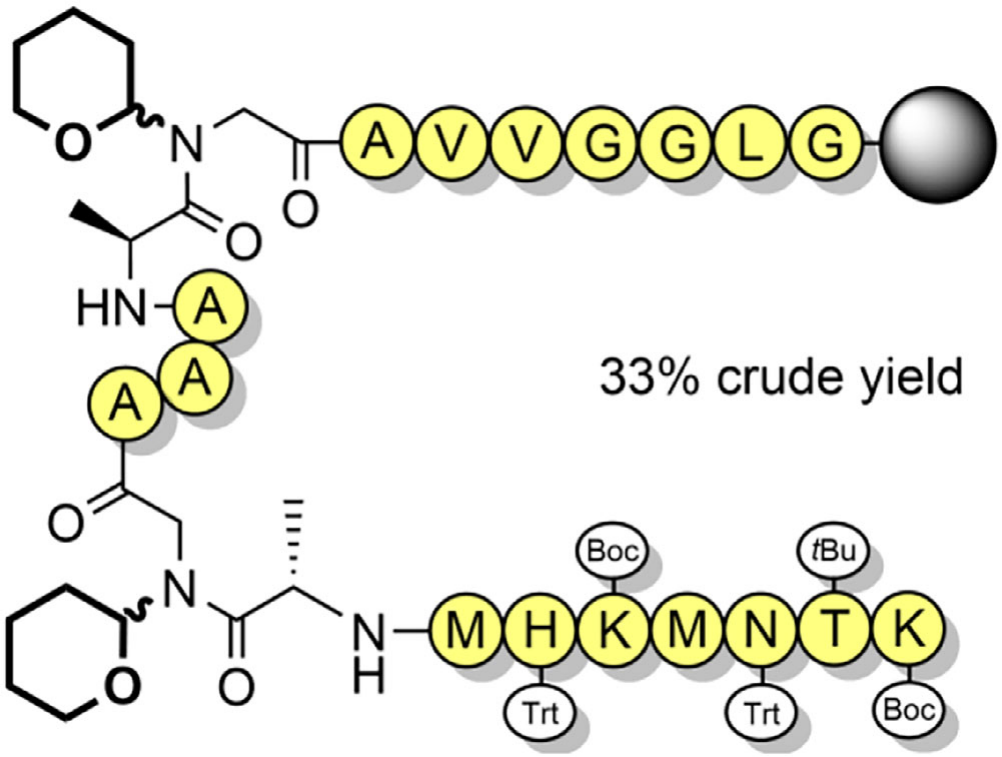
Synthetic strategy for the efficient preparation of PrP(106–126), utilizing two Thp backbone protecting groups.

The tetrahydropyranyl (Thp) group has been evaluated as a more acid labile alternative to benzyl protection (Figure 2l).^[^ ^169^ ^]^ The Thp group can be efficiently incorporated into the resin‐bound peptide as a protected dipeptide, and is readily cleaved and scavenged postsynthesis. Synthesis of the protected alanine‐ and glycine‐containing dipeptides proceeds first through acid‐catalyzed alkylation of amino acid benzyl esters with 3,4‐dihydro‐2*H*‐pyran. Coupling of Fmoc amino acids onto the hindered amine is challenging, but can be achieved in good yields via the mixed anhydride method in the presence of *N*,*O*‐bis(trimethylsilyl)acetamide as an amine‐activating additive;^[^ ^170^ ^]^ hydrogenolysis of the benzyl ester then generates the Thp‐protected dipeptide acid (Scheme 17). A substantial improvement in the solid‐phase assembly of aggregation‐prone amyloid‐β and prion‐derived peptide fragments is observed using Thp backbone protection (Figure 7). Thp‐protected dipeptides have potential as useful building blocks for efficient peptide synthesis with further optimization, complementing existing backbone protecting group strategies.

### 2.6 Dicyclopropylmethyl Protection

**Scheme 18:**
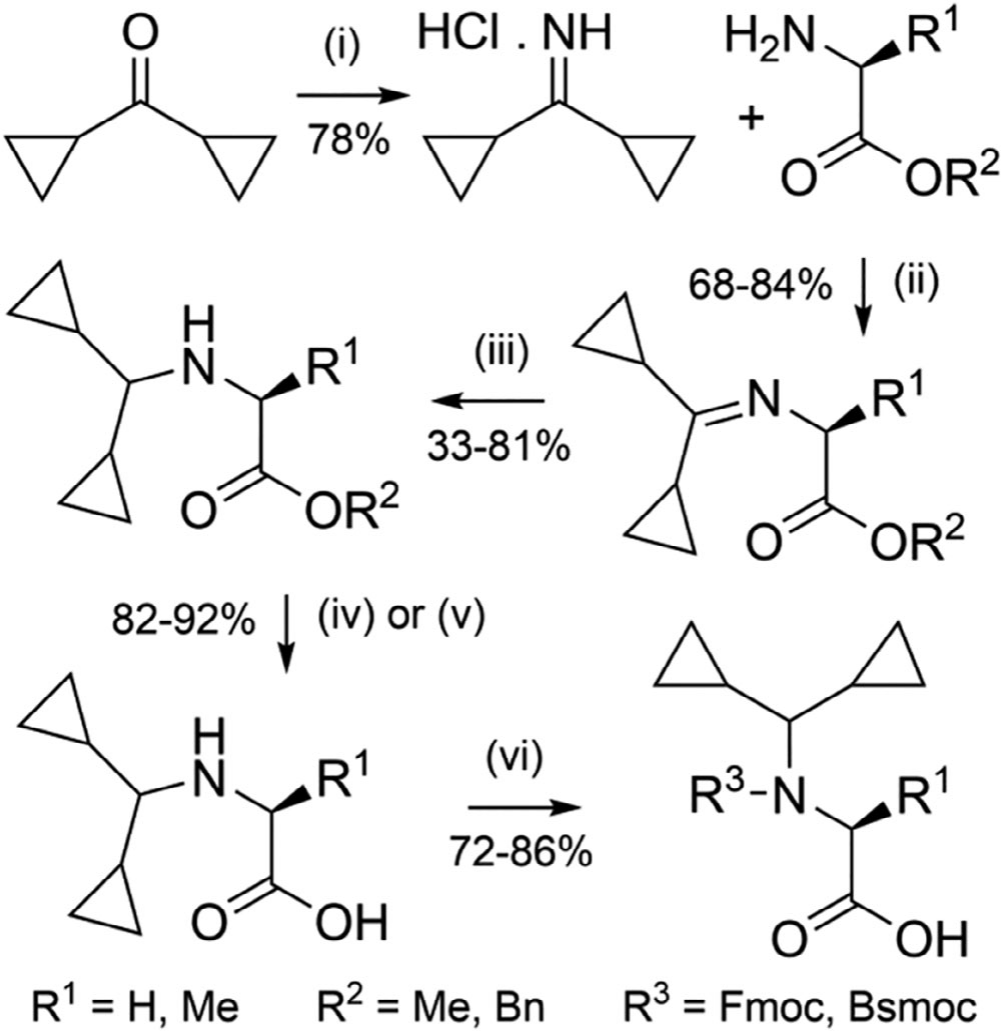
Synthesis of Dcpm‐protected Fmoc amino acids. i) TiCl_4_, NH_3_, benzene, HCl. ii) TEA, CH_2_Cl_2_. iii) NaBH(OAc)_3_. iv) Hydrolysis of methyl esters with NaOH. v) Hydrogenolysis of benzyl esters with H_2_ and Pd/C. vi) Trimethylsilyl chloride, CH_2_Cl_2_, N_2_, DIEA, Fmoc‐Cl or benzo[*b*]thiophenesulfone‐2‐methyloxycarbonyl chloride (Bsmoc‐Cl).

**Figure 8:**
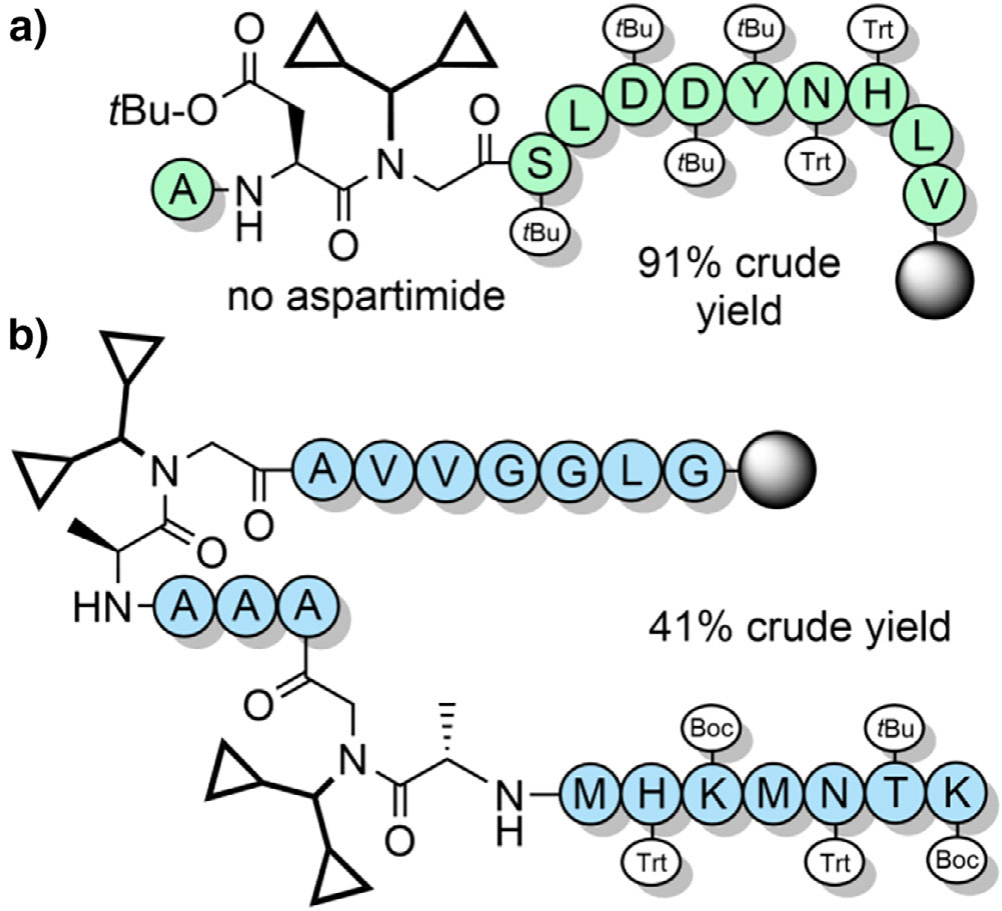
Application of Dcpm backbone protection. a) Aspartimide‐free synthesis of an Asp‐Gly‐containing peptide. b) Efficient synthesis of PrP(106–126) with two Dcpm groups.

The Dcpm (Figure 2m) and dimethylcyclopropyl (Dmcp) groups have been investigated for their high acid lability, which is attributed to the increased stability of cyclopropylmethyl‐based cations.^[^ ^171^ ^]^ Dcpm can be introduced onto the solid support through Fmoc‐amino acid building blocks, which can be accessed efficiently from dicyclopropylmethanimine (Scheme 18).^[^ ^94^ ^]^ However, Dmcp‐protected amines contain a tertiary carbon which makes acylation inefficient due to steric hindrance. This limits its use as a backbone protecting group to alanine and glycine residues. Dcpm has been used to prevent aspartimide formation through the use of Fmoc‐Asp(O*t*Bu)‐(Dcpm)Gly‐OH dipeptide building blocks, which avoids a difficult coupling onto the hindered *N* ^α^‐amine on the solid support (Figure 8a).^[^ ^100^ ^]^ Its impact on peptide assembly was determined using PrP(106–126) as a model system (Figure 8b), where crude purity was found to be 41%. This compares favorably with the corresponding synthesis using Hmb backbone protection, which resulted in only 7% crude purity.^[^ ^94^ ^]^ It is somewhat surprising that the Dcpm group is not more widely used for glycine and alanine, given its effectiveness in suppressing aggregation, and its high acid lability.

### 2.7 Photocleavable Protecting Groups

**Scheme 19:**
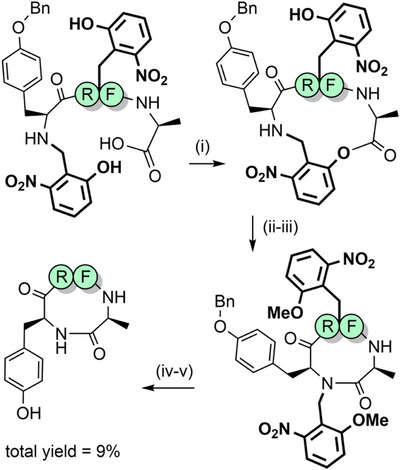
The use of 2‐nitrobenzyl‐based backbone protection. i) Cyclization. ii) *O*→*N* acyl shift. iii) CH_2_N_2_. iv) UV, 365 nm. v) H_2_/Pd.

2‐Hydroxynitrobenzyl‐based protecting groups have also been evaluated (Figure 2n).^[^ ^172^ ^]^ The primary reason for their use was to increase the rate of *O*→*N* acyl migration for hindered dipeptide junctions, to broaden the utility of backbone protection. The electron withdrawing nitro group improves the leaving group character of the *O*‐aryl group and simultaneously increases activation of the carbonyl carbon of the incoming Fmoc‐amino acid. The 2‐hydroxy‐5‐nitrobenzyl (2,5‐Hnb) and the 2,6‐Hnb groups were found to be superior acyl transfer auxiliaries compared with Hmb. Notably, both 2,5‐Hnb and 2,6‐Hnb groups enabled synthesis of the highly hindered valine–valine unit, in >90% yield. These protecting groups are TFA stable, but only the 2,6‐Hnb group—where the nitro group is in the ortho position—can be removed via photolysis (at 366 nm).^[^ ^173^ ^]^ 2,5‐Hnb is not photolabile and therefore of limited utility unless modified further (see Section 2.9). 2,6‐Hnb has also been investigated as an N‐terminal cyclization auxiliary for short peptides.^[^ ^174^, ^175^ ^]^ Macrocyclization proceeds via esterification between the accessible phenolic group of Hnb and the C‐terminus, followed by ring contraction through *O*→*N* acyl migration and finally photocleavage of the Hnb auxiliary (Scheme 19). Larger ring sizes are more synthetically accessible, as demonstrated in the synthesis of several backbone‐cyclized somatostatin analogues.^[^ ^176^, ^177^, ^178^ ^]^ The 4‐methoxy‐2‐nitrobenzyl group (Figure 2o) can also be introduced into the peptide backbone for improving assembly, although at less hindered amide junctions. It can be photolytically cleaved in approximately 2 h, using cysteine as a scavenger to trap the benzaldehyde side product.^[^ ^179^ ^]^ Given its TFA stability, 2‐nitrobenzyl‐based backbone protection can also be utilized in Boc SPPS.

### 2.8 Propargyl Protection

**Scheme 20:**
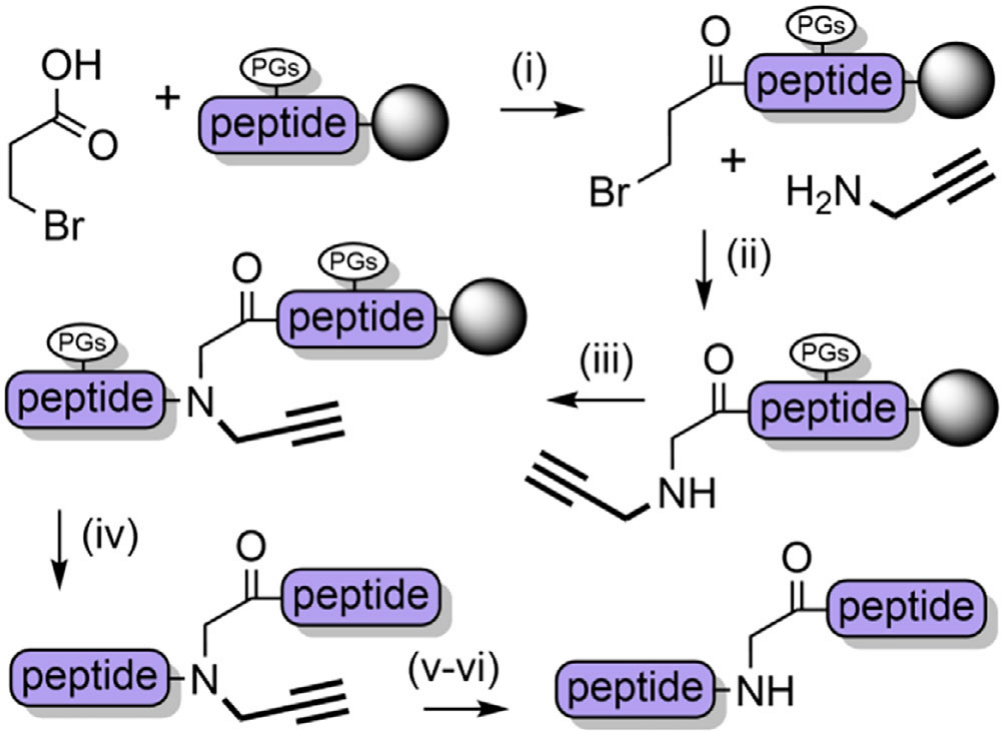
Propargyl backbone protection. i) DIC, DMF, 40 m. ii) DMF, 40 m. iii) Fmoc SPPS. iv) 2.5% TIPS, 2.5% H_2_O, 95% TFA, 1.5 h. v) 6 M guanidinium hydrochloride, 200 mM Na_2_HPO_4_, pH 7.3, 2 mM, 20 equiv AuCl, 42 °C. vi) Dithiothreitol.

The retention of backbone protecting groups on peptides postcleavage can suppress aggregation in solution and improve NCL yields. To this end, the propargyl group (Prop) (Figure 2p)—which is sterically small—has been investigated as a TFA‐stable *N*‐protecting group.^[^ ^180^ ^]^ Introduction of the *N*‐propargylglycyl moiety to resin‐bound peptides is analogous to the sub monomer method that is used to assemble peptoids,^[^ ^181^ ^]^ and is therefore operationally simple. In short, the N‐terminus is first acylated with bromoacetic acid under carbodiimide‐mediated base free conditions. Propargylamine is then introduced to form the secondary amine via an S_N_2 reaction, followed by conventional Fmoc SPPS (Scheme 20). Depropargylation at a range of Xaa‐(Prop)Gly junctions is achieved in 80%–95% yield, using gold(I) chloride which coordinates to the alkyne. *N*‐Propargyl backbone protection was applied to a single‐shot synthesis of the ca. 8.5 kDa NEDD8 protein.^[^ ^182^ ^]^ The product was obtained in 45% isolated yield, albeit with approximately 10% cleavage of the amide at the propargylation site. This side reaction—which is the major product for Gly‐(Prop)Xaa junctions (70%–95%)—was exploited for use as a mild and rapid cleavage mechanism to release a biotin‐tagged peptide from a streptavidin‐coated plate. Backbone *N*‐propargylated unprotected peptides can also be cyclized (at the N‐terminus) under mild conditions using gold(I),^[^ ^183^, ^184^ ^]^ thus highlighting the versatility of this chemistry. Further investigation of the *N*‐propargyl group and its reactivity with a range of transition metals is certainly warranted.

### 2.9 Safety‐Catch Protecting Groups

**Scheme 21:**
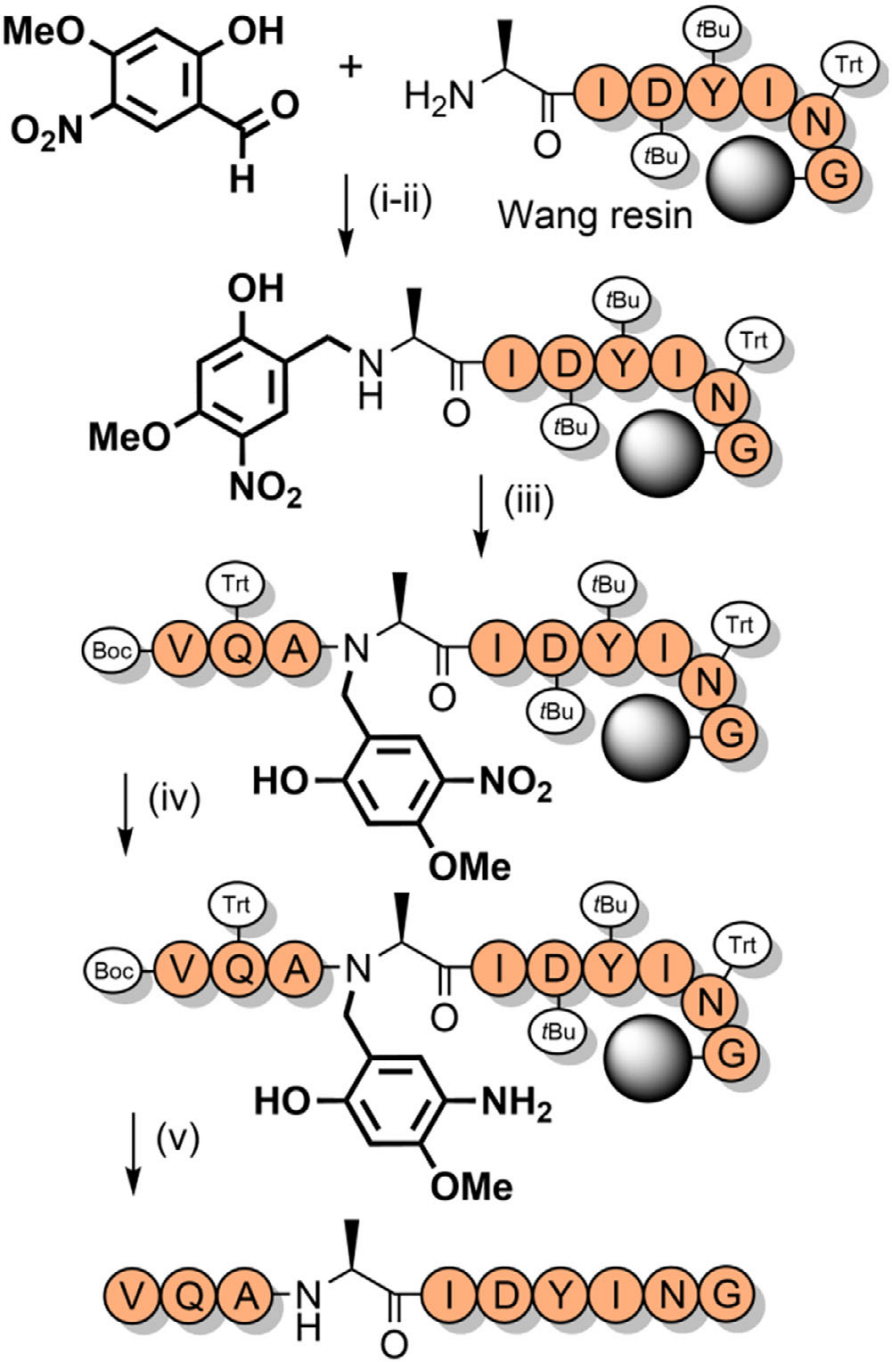
Nitrobenzyl‐based backbone protection with tunable acid lability. i) 2‐Hydroxy,4‐methoxy‐5‐nitrobenzaldehyde, DMF. ii) NaBH_4_, DMF. iii) Fmoc SPPS. iv) CrCl_2_, DMF, 70 °C, 2 h. v) TFA (85%), trimethylsilyl bromide (9%), thioanisole (4%), 1,2‐ethanedithiol (2%), 1 h.

“Safety‐catch” groups—often incorporated as linkers between the resin and the peptide C‐terminus—are stable to peptide elongation conditions, but upon modification of their functional groups are rendered more labile to acid catalyzed cleavage. Such tunable groups have also been developed as peptide backbone protecting groups to enable their temporary retention after TFA cleavage. 2‐Hydroxy‐4‐methoxy‐5‐nitrobenzyl (Hmnb)—which is electron deficient—is one such protecting group (Figure 2q). Its presence in the peptide backbone can enhance solubility and suppress aggregation during solution‐phase handling and purification. The aryl nitro group can then be reduced to an electron‐donating amine, making it TFA‐labile.^[^ ^185^ ^]^ Hmnb has the advantage of facilitating superior coupling kinetics over Hmb due to the electron withdrawing nitro group increasing the rate of the *O*→*N* acyl transfer (as per 2,5‐Hnb and 2,6‐Hnb).^[^ ^185^, ^186^ ^]^ This has enabled the introduction of backbone protecting groups at sterically hindered sites. Hmnb has been used for the synthesis of a polyalanine peptide (86% yield), and the efficient assembly of ACP(65–74) (Scheme 21). It is also effective at suppressing aspartimide formation when using sterically small aspartyl protecting groups that favor succinimidyl formation, such as the *O*‐allyl and *O*‐2‐(*tert*‐butyldisulfanyl)ethyl groups.^[^ ^187^ ^]^ Unfortunately, even when the aryl nitro group of Hmnb is reduced to the aniline, TFA cleavage is still significantly slower compared to Hmb. However, this problem can be circumvented via diazotization of the aryl amine and then elimination, to ultimately generate Hmb.^[^ ^188^ ^]^

**Scheme 22:**
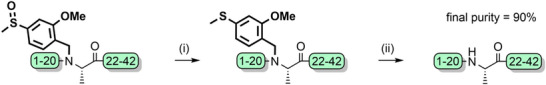
Application of thioether/sulfoxide‐based backbone protection with tunable acid lability for the synthesis of Aβ(1–42). i) NH_4_I. ii) TFA.

Sulfoxide‐containing “safety catch” protecting groups have also been developed, which are analogous to nitro‐containing groups. While the sulfoxide‐containing group is TFA‐stable, reduction to the electron‐donating sulfide increases acid lability.^[^ ^186^ ^]^ The reduction can be conducted just prior to global deprotection to remove it, or after purification. Examples include the 6‐hydroxy‐1,3‐benzoxathiole,^[^ ^186^ ^]^ 3‐methylsulfinyl‐4‐methoxy‐6‐hydroxybenzyl,^[^ ^189^ ^]^ and 2‐methoxy‐4‐methylsulfinylbenzyl (Mmsb)^[^ ^87^ ^]^ groups (Figure 2r). The Mmsb group was used to synthesize Aβ(1–42) in 35% crude yield, with retention of the sulfoxide to improve the peptide's solubility during characterization and purification. Reduction followed by cleavage furnished the target peptide in 90% purity (Scheme 22).^[^ ^87^ ^]^

**Scheme 23:**
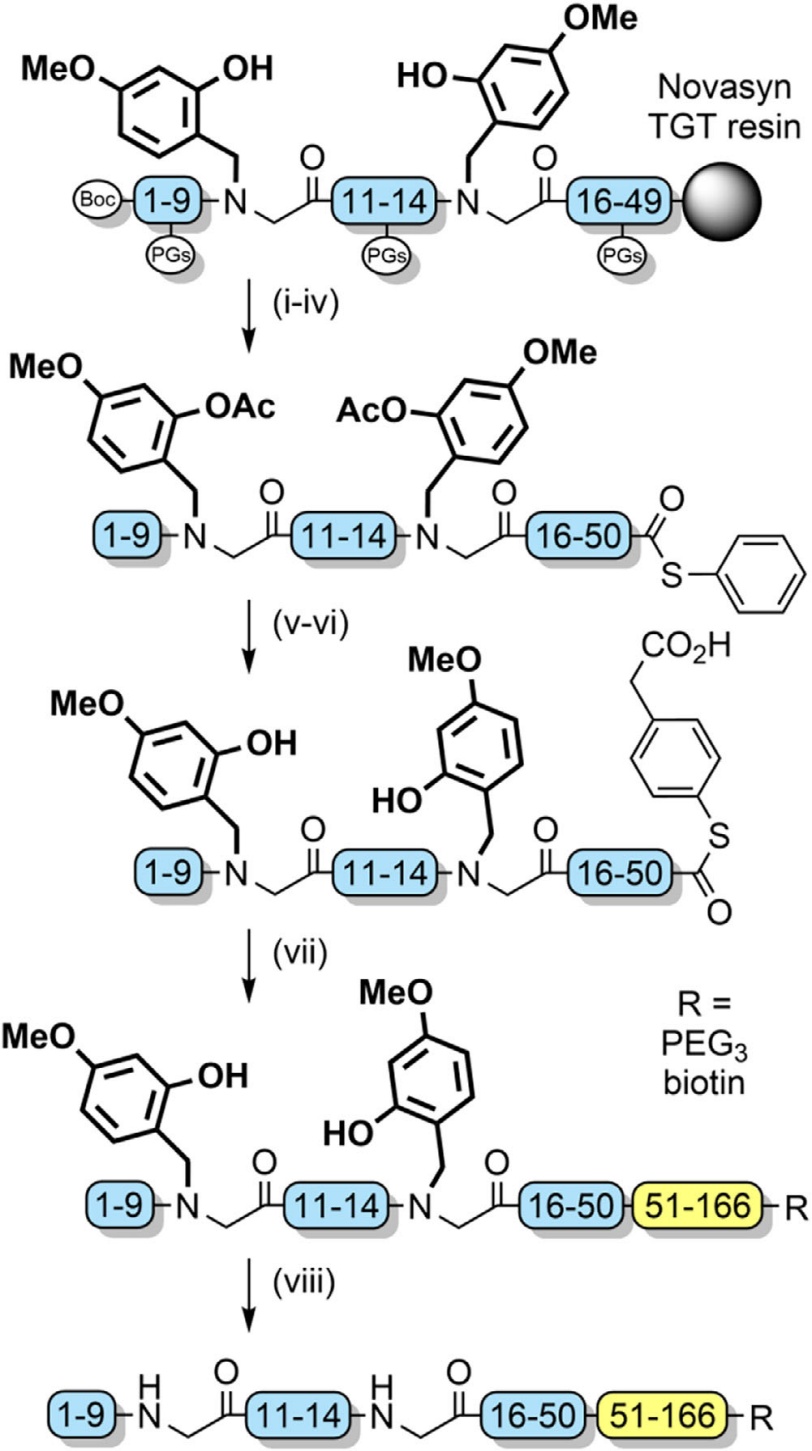
Acetoxybenzyl groups for the preparation of K‐Ras. i) Ac_2_O, DIEA, DMF. ii) CH_2_Cl_2_ (80%), TFE (10%), acetic acid (10%). iii) EDC, HOBt, H‐Thr‐SPh·HCl. iv) TFA, TIPS, H_2_O, phenol. v) Guanidinium hydrochloride/Na_2_HPO_4_ buffer. vi) MPAA, *tris*(2‐carboxyethyl)phosphine·HCl (TCEP·HCl), pH 6.8. vii) Guanidinium hydrochloride/Na_2_HPO_4_/TCEP·HCl buffer, pH 7.0. viii) TFA (90%), thioanisole (5%), 1,2‐ethanedithiol (3%), anisole (2%).

2‐Acetoxy‐4‐methoxybenzyl (AcHmb) is an easily accessible TFA‐stable analogue of Hmb (Figure 2s). The increased acid stability is due to the 2‐acetoxy group having a reduced electron donating effect compared with a hydroxyl group. AcHmb is deacetylated with 20% piperidine; therefore, *O*‐acetylation of incorporated Hmb groups must be conducted after peptide assembly, to enable retention of the protecting group after TFA cleavage. AcHmb then exerts its solubilizing effect throughout multiple solution‐phase steps, with deacetylation just prior to final TFA treatment to enable its removal.^[^ ^190^ ^]^ This method was successfully applied to the synthesis of the N‐terminal hydrophobic segment of K‐Ras GTPase (Scheme 23). After protein assembly via NCL, the Hmb groups were cleaved via a TFA cocktail to obtain the native 166‐residue protein in 19% isolated yield.^[^ ^13^ ^]^

**Scheme 24:**
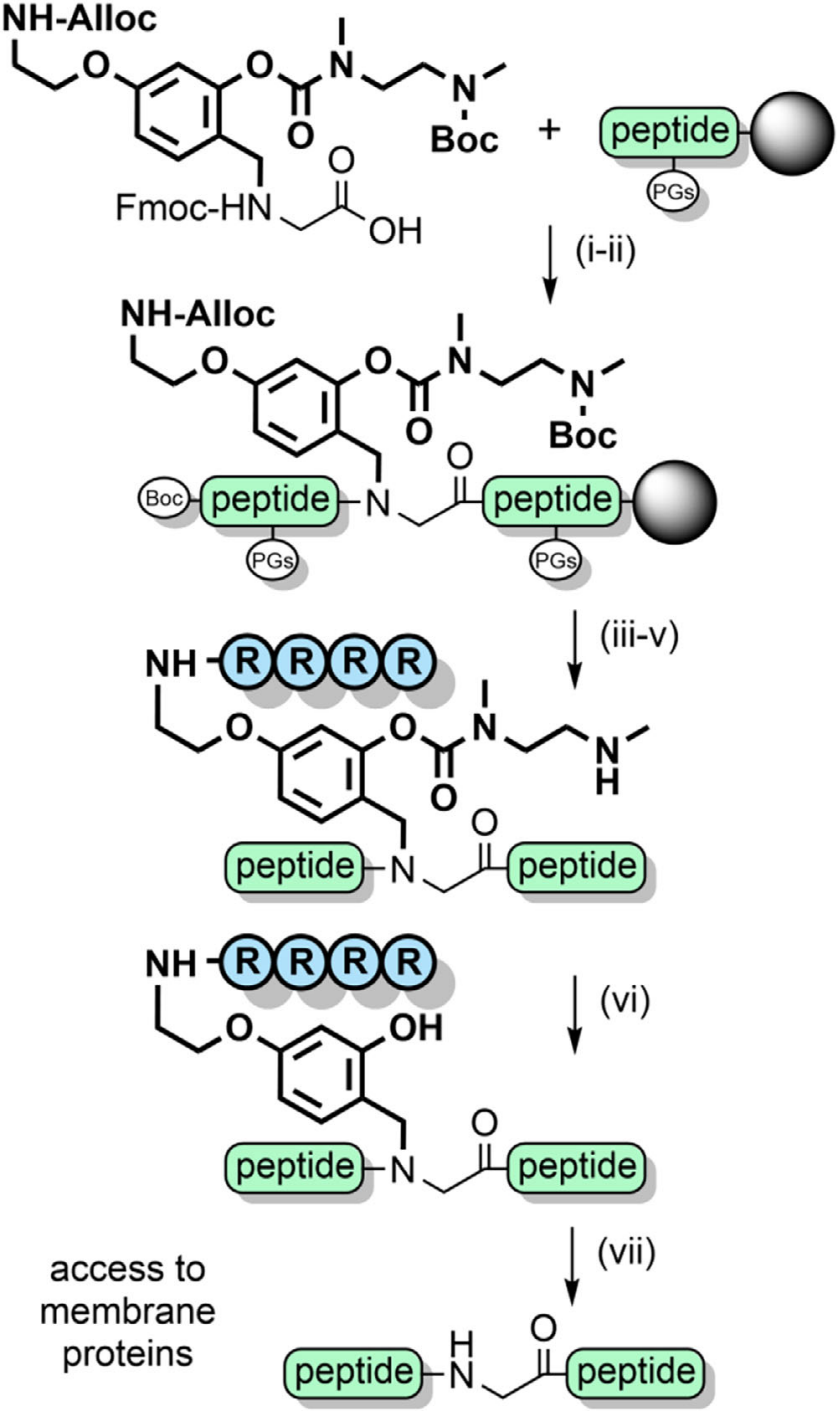
Synthesis of membrane proteins through a pH‐sensitive tunable backbone protecting group. i) Fmoc SPPS. ii) 20% piperidine in DMF. iii) Pd(PPh_3_)_4_, PhSiH_3_. iv) Fmoc SPPS with arginine. v) TFA. vi) Neutral buffer. vii) TFA.

**Scheme 25:**
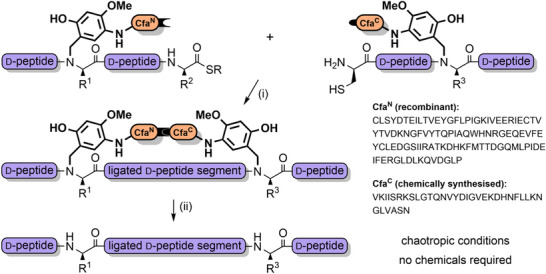
Backbone installed split intein‐assisted ligation (BISIAL). i) 8 M urea, 0.1 M Na_2_HPO_4_, 150 mM NaCl, 2 mM TCEP·HCl, 1 mM EDTA, pH 7.2. ii) 0.1 M HCl, 1% TIPS, 1,1,1,3,3,3‐hexafluoro‐2‐propanol, 30 °C, 1 h.

**Scheme 26:**
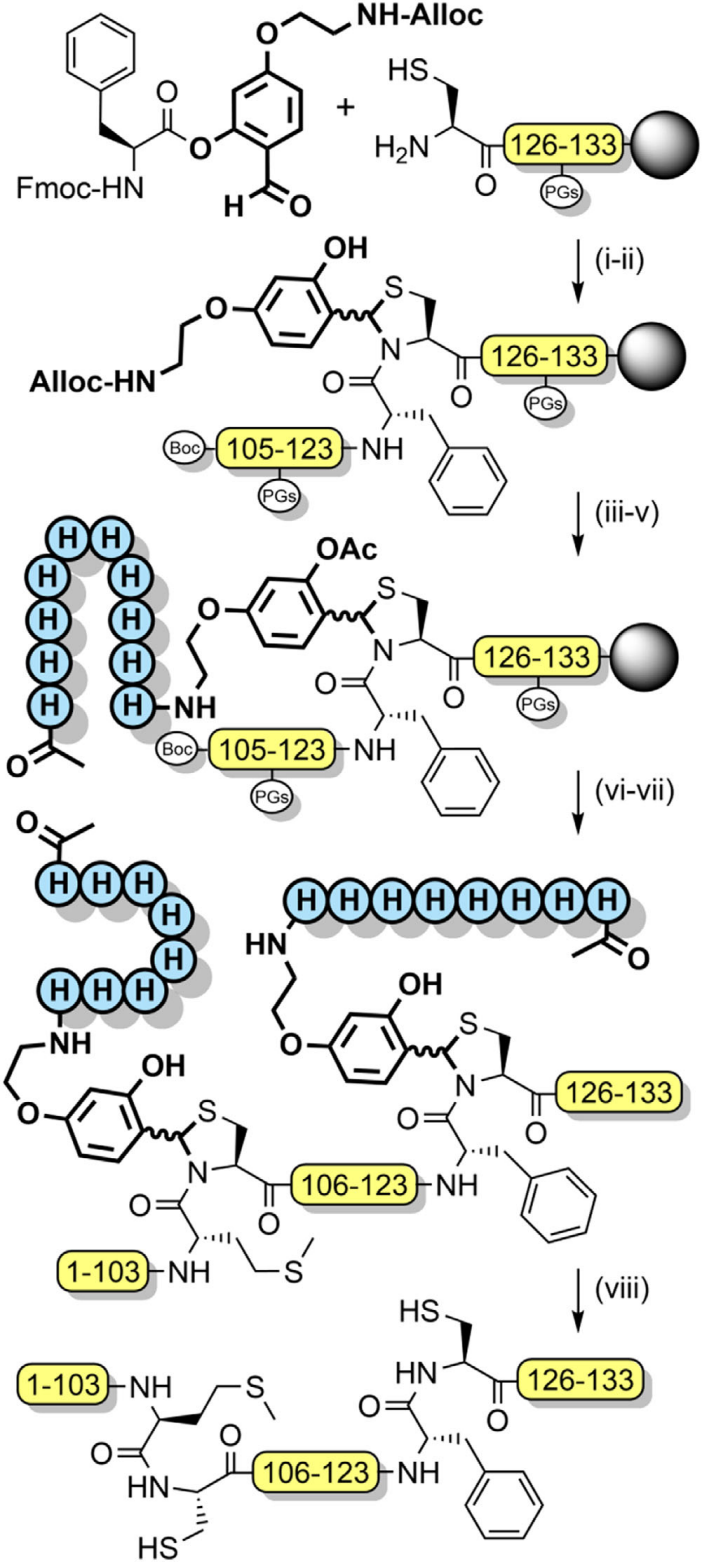
*S*,*N*‐Benzylidene‐based tunable backbone protection for the assembly of aggregation‐prone proteins. i) acetic acid, pyridine. ii) Fmoc SPPS, HATU, DIEA. iii) Pd(PPh_3_)_4_, PhSiH_3_. iv) Fmoc SPPS with histidine. v) Ac_2_O, TEA. vi) TFA (95%), TIPS (2.5%), H_2_O (2.5%). vii) Peptide hydrazide, NaNO_2_, pH 3, 4‐mercaptophenylacetic acid, then pH 6.5. viii) TFA (92.5%), TIPS (2.5%), 1,2‐ethanedithiol (2.5%), H_2_O (2.5%).

AcHmb has also been used to prevent aspartimide formation, and improve peptide solubility during the synthesis of glycopeptides^[^ ^191^ ^]^ It is effective in inhibiting the formation of peptide aggregates such as soluble colloidal particles that reduce the efficiency of NCL.^[^ ^192^ ^]^ *O*‐Acylated Hmb with a pH‐sensitive “switch” has also been developed, which functions via an intramolecular *O*→*N* acyl shift.^[^ ^193^, ^194^ ^]^ Further solubility enhancements were achieved by appending poly‐arginine sequences to the backbone benzyl group, via an aminoethoxy linkage (Scheme 24).^[^ ^195^ ^]^ Solubilizing tags can also be introduced via aniline, which can be obtained through reduction of the nitro group of Hmnb; TFA lability can still be controlled through *O*‐acylation of the 2‐hydroxyl group.^[^ ^196^, ^197^ ^]^ Other NCL strategies also exploit the aryl amino group as a reactive handle. For example, the N‐ and C‐exteins of the unique consensus‐fast split intein—which rapidly associate—were each appended to cleavable backbone protecting groups on two separate peptide ligation partners. After extein ligation, NCL of the peptide fragments is rapid due to proximity effects (Scheme 25). This method is particularly useful at high dilution, for hindered ligation sites and suitable for the preparation of membrane proteins.^[^ ^198^ ^]^ *N*,*S*‐Benzylidenes bearing an Alloc‐protected aminoethoxy moiety have also been utilized to introduce prosthetic groups.^[^ ^144^ ^]^ After chain assembly, orthogonal Alloc deprotection enabled installation of a poly‐histidine solubilizing tag with *O*‐acetylation preserving the prosthetic group during TFA deprotection. The solubilizing tag and the “kinked” backbone enabled the efficient assembly of the aggregation‐prone hydrophobic C‐terminal region of interleukin‐2 (Scheme 26).^[^ ^199^ ^]^

## 3 Discussion

Backbone protection has become a vital strategy for accessing aggregation prone peptides and small proteins on the solid support. Hmb^[^ ^89^, ^90^, ^95^ ^]^ and Dmb^[^ ^83^, ^84^, ^109^ ^]^ are commonly used, with several building blocks commercially available. However, Dmb can only be introduced efficiently at glycine‐containing dipeptide junctions due to steric hindrance. The Hmb group overcomes these steric effects through *O*‐acyl capture. This acyl transfer process has underpinned the development of a range of commercially available Hmb monomers and dipeptides. However, Hmb protection is not well‐suited for large‐scale peptide synthesis, wherein only 1.5–2.0 equiv of amino acid are typically used, to minimize manufacturing costs. This is because the aryl 2‐hydroxyl group can compete with the N‐terminus in coupling reactions, which depletes the amino acid reactant and can lead to incomplete coupling. This issue does not impact small scale synthesis as 5–10 equiv of amino acid are typically added. Commercially available serine‐, threonine‐, and cysteine‐based pseudoproline‐containing amino acids and dipeptides have revolutionized Fmoc SPPS and can be used on any scale,^[^ ^92^, ^122^ ^]^ including large‐scale (e.g., liraglutide^[^ ^118^ ^]^). They are produced efficiently and are very effective in improving chain solubility and disrupting β‐sheets. Iso‐acyl dipeptides—also commercially available—have the additional advantage of improving handling in solution.^[^ ^145^, ^147^, ^154^, ^156^ ^]^ More recently, *N*,*O*‐benzylidene acetal dipeptides have been shown to further improve the quality of crude peptide products.^[^ ^143^ ^]^ However, these backbone protecting group strategies are limited to serine, threonine, and cysteine‐rich peptides, and cannot be used universally. A range of novel heterocyclic,^[^ ^90^, ^164^ ^]^ alkyloxymethyl,^[^ ^168^ ^]^ and Dcpm^[^ ^94^ ^]^ *N*‐protecting groups have also been investigated. Unfortunately, none of these examples have found widespread use, which is partly due to poor yields for their incorporation and/or suboptimal deprotection kinetics. The development of new hyper‐labile backbone protecting groups that can be introduced within aliphatic‐rich peptide segments (such as transmembrane domains) would be of great value to the field of peptide chemistry.

Backbone protection can also suppress peptide aggregation in solution, which has long hampered purification. Aggregation prone sequences are often insoluble and fail to elute as symmetrical peaks on reversed‐phase HPLC. This leads to peak overlap with peptidic impurities, and ultimately low purity products and poor yields. Iso‐acyl dipeptides have been instrumental in addressing this aggregation problem, most notably for Aβ(1–42)^[^ ^154^ ^]^ and insulin.^[^ ^159^, ^160^ ^]^ A range of TFA‐stable benzylic groups bearing safety‐catch functionalities have also been developed for improved solution‐phase handling of peptides. After purification, acid lability is conveniently restored via conversion of electron withdrawing substituents to electron donating ones (e.g., nitro→amine,^[^ ^185^, ^186^ ^]^ sulfoxide→thioether,^[^ ^87^, ^186^, ^189^ ^]^ and acetoxy→hydroxy^[^ ^13^, ^144^, ^190^, ^191^, ^192^, ^193^, ^194^, ^198^, ^199^ ^]^). Safety‐catch groups that enable temporary backbone protection have been particularly effective for NCL,^[^ ^194^ ^]^ which is often plagued by poor yields due to aggregation, even in denaturing buffers. Peptide and protein solubility has been further augmented by appending hydrophilic tags to the protecting group.^[^ ^144^ ^]^ These auxiliaries are now used routinely for chemical protein synthesis and have enabled access to a range of challenging targets well over 100 amino acids in length.

Another important application of backbone protection is peptide macrocyclization. The inherent linearity of peptide chains is unfavorable for forming macrocycles, but the introduction of backbone protection induces a *cis*‐amide conformation that promotes cyclization.^[^ ^135^ ^]^ Peptide macrocyclization can also be achieved on the solid support through backbone anchoring,^[^ ^116^ ^]^ or through acyl capture via N‐terminal backbone auxiliaries.^[^ ^174^ ^]^ Moreover, a range of fragment couplings can now be conducted epimerization free through the use of backbone protection.^[^ ^85^, ^139^, ^162^ ^]^ Aspartimide formation still bedevils peptide chemistry,^[^ ^114^ ^]^ particularly with the increase in use of heat‐assisted Fmoc SPPS. The use of bulkier aspartyl side chain protecting groups can minimize this side reaction,^[^ ^200^ ^]^ but backbone protection blocks it completely. Use of the commercially available Fmoc‐Asp‐(Dmb)Gly‐OH building block is essential, and Hmb use is recommended for Asp–Ala, Asp–Asn, and Asp–Asp dipeptide junctions. Aspartimide formation at Asp–Ser, Asp–Thr, and Asp–Cys junctions can be avoided through the use of pseudoproline dipeptides.

Despite the numerous backbone protecting groups that have been developed for Fmoc SPPS, each strategy has its limitations. Therefore, it is prudent to summarize the existing protecting groups (Table 2) as a guide to assist researchers in understanding the most suitable approaches for using these techniques, and to ignite further interest in this field of research.

**Table 2:** Summary of the backbone protecting groups that have been developed to date for Fmoc SPPS.

Protecting group	Advantages	Disadvantages	Refs.
Hmb^a)^ (Figure 2a)	Can be used “universally” Can be introduced in situ	Reactive hydroxyl group Cleavage often inefficient	[89, 90, 97]
Dmb^a)^ (Figure 2b)	Can be introduced in situ Blocks aspartimide for DG	Largely limited to Gly Cleavage often inefficient	[2, 63, 80, 83, 109]
Tmb (Figure 2c)	Excellent lability Backbone anchoring	Largely limited to Gly	[90]
Pseudoprolines^a)^ (Figure 2d)	Dual *O*‐ and *N*‐protection Promotes cyclizations	Limited to Ser, Thr, and Cys	[91, 92, 93, 119, 122]
*N*,*X*‐Benzylidenes^b)^ (Figure 2e,f)	“Kinked” backbone further enhances peptide assembly	Limited to Ser, Thr, and Cys Reactive hydroxyl (for *N*,*O*)	[143, 144]
Isoacyl^a)^ (Figure 2g)	Solution handling improved Epimerization‐free couplings	Limited to Ser and Thr Side reactions possible	[145, 147, 154]
Furfuryl/thienyl‐methyl (Figure 2h)	Less sterically hindered than benzyl‐based groups	Poor synthetic yields	[90]
EDOTn/MIM (Figure 2i,j)	Excellent acid lability	No substantial advantages over benzyl groups	[164]
Etom (Figure 2k)	High acid lability	Low yield of precursors Alloc protection required	[168]
Thp (Figure 2l)	High acid lability	Stereoisomeric precursors Largely limited to Gly and Ala	[169]
Dcpm (Figure 2m)	High acid lability	Largely limited to Gly	[94]
Nitrobenzyl (Figure 2n,o)	Increased acylation kinetics Can be used “universally”	UV‐reactor required	[172, 174]
Propargyl (Figure 2p)	Simple to introduce Solution handling improved	Largely limited to Gly Amide cleavage side reaction	[180, 182, 183, 184]
Hmnb^a)^ (Figure 2q)	Increased acylation kinetics Can be used “universally”	Not compatible with disulfide rich peptides	[185, 186]
Mmsb (Figure 2r)	Tunable TFA reactivity	Not compatible with disulfide rich peptides	[87]
AcHmb^a)^ (Figure 2s)	Tunable TFA reactivity Solution handling improved	An additional step is required to enable TFA stability	[190]

^a)^ Commercially available precursors or reactants.

^b)^ X = *O*, *S*.

## 4 Future Outlook

The increasing demand for synthetic peptides and proteins—which has been largely driven by the rapid growth of peptide therapeutics^[^ ^35^, ^36^, ^37^, ^38^ ^]^—has necessitated improvements in peptide manufacturing for both small‐scale and cGMP synthesis. To this end, the development of SPPS has been crucial for enabling rapid access to peptide analogues, including mirror image peptides and proteins, which can only be prepared via chemical methods.^[^ ^201^ ^]^ The advent of Fmoc SPPS (which uses “mild” reagents) and automation has also been a democratizing force by allowing laboratories without specialized knowledge to acquire a peptide synthesis capability. However, the iterative nature of SPPS and the inherent property of peptides and proteins to self‐assemble leads to high synthetic failure rates for longer peptides, which significantly hampers peptide‐based discovery science and drug development. Therefore, there is a need to further improve Fmoc SPPS, such as through the use of backbone protection. Consistent access to chemically diverse peptides >40 amino acids in length would be ideal—for both research applications and large‐scale cGMP synthesis—such that chemical methods become even more competitive with recombinant protein synthesis. The routine preparation of larger peptide fragments will also expedite protein assembly via NCL, as fewer ligations will be required to access the desired target.

For cGMP SPPS‐based manufacturing, there are strict purity tolerances compared to research‐grade peptides. Therefore, it is crucial to investigate the use of the appropriate protecting groups early in the process design phase, as this impacts the impurity profile. High crude purities are essential to ensure that analytical and, most importantly, large‐scale preparative chromatographic methods can be established to separate the impurities from the API. Health authorities are intensifying their focus on impurity profiles and have thus tightened the regulatory framework relating to API manufacturing. This added regulatory burden necessitates the need for further improvement in peptide synthesis methodology, such as through the development of new backbone protecting groups to minimize the formation of structurally similar, intractable impurities.

Heat‐assisted^[^ ^51^, ^202^ ^]^ and continuous flow^[^ ^31^, ^57^, ^59^ ^]^ automated SPPS have reduced the synthetic failure rate to a significant degree and enabled access to longer targets that can be obtained via single‐shot synthesis. However, backbone protection addresses the fundamental solubility and aggregation problems of peptides and proteins both on the solid support and in solution. Therefore, methods to automate the introduction of backbone protecting groups cost‐effectively are highly desirable. One notable effort toward this goal involves the use of a flow peptide synthesizer to efficiently *N*‐acylate hindered N‐terminal pseudoproline monomers on the solid support, which are more versatile and less expensive than pseudoproline dipeptides.^[^ ^123^ ^]^ Moreover, Dmb can be introduced via flow chemistry to suppress aspartimide formation, which occurs more frequently due to the higher temperatures used.^[^ ^59^ ^]^ The introduction of novel benzyl‐based protecting groups through reductive amination can also be automated.^[^ ^203^ ^]^

Pseudoproline and iso‐acyl dipeptides will undoubtedly continue to be the first choice for introducing backbone protection into peptides.^[^ ^92^, ^122^ ^]^ The cost of these building blocks has declined somewhat, and they are now available from several vendors. *N*,*O*‐Benzylidene acetal dipeptides appear to provide further enhancement in peptide assembly and may be utilized more frequently in future.^[^ ^143^ ^]^ The key disadvantage of all these precursors is their limited utility, as they can only be introduced at serine, threonine, and cysteine residues, which represents <14% of amino acid natural abundance.^[^ ^204^ ^]^ Benzyl‐based protecting groups can be used more universally, which is useful for aliphatic‐rich segments that are particularly prone to aggregation. However, their acid lability can depend on the nature of the flanking amino acid side chains and is sequence dependent with incomplete deprotections possible. Alkylation of cysteine, methionine, and tryptophan residues by liberated benzyl cations can also occur. Therefore, the use of numerous benzyl groups for a single synthesis is not recommended. These are limiting factors for Fmoc SPPS, as ideally the incorporation of multiple backbone protecting groups will enable routine access to peptides and proteins well in excess of 40 residues in good yield.^[^ ^61^ ^]^

New highly acid‐labile backbone protecting groups that can be efficiently and universally introduced are therefore needed to address the limitations described above. Dcpm is one protecting group that is perhaps underutilized. Although its steric bulk largely limits its use to glycine (still at >7% natural abundance) and possibly alanine, it is readily cleaved in TFA and thus avoids the deprotection problem.^[^ ^94^ ^]^ *N*,*O*‐Acetals are similarly acid labile and could become more viable with improvements in their synthesis.^[^ ^168^, ^169^ ^]^ Reinvestigation of nitrobenzyl‐based protecting groups is also warranted given that they can be introduced at hindered sites.^[^ ^172^ ^]^ Moreover, these versatile photocleavable auxiliaries can be used to improve handling in solution. Smaller electron‐rich 5‐membered heterocycles are another avenue worthy of investigation.^[^ ^90^ ^]^ Further optimization of backbone protecting groups, in combination with complementary peptide chain solubilizing strategies such as cleavable polycationic tags^[^ ^76^, ^205^, ^206^, ^207^ ^]^ and polar TFA‐stable *S*‐protecting groups,^[^ ^208^, ^209^, ^210^ ^]^ promises more routine access to challenging synthetic targets such as membrane proteins. These advances, together with the latest iteration of peptide synthesizers^[^ ^211^ ^]^ and state‐of‐the‐art chromatographic separation technologies,^[^ ^212^ ^]^ will be instrumental in extending the capabilities of Fmoc SPPS.

## 5 Conclusion

Backbone protection is a proven strategy for improving peptide and protein chemical synthesis. Longer peptides, small proteins, and aggregation‐prone sequences rich in aliphatic amino acids can now be assembled with much greater efficiency. Backbone protection also improves the synthesis of cyclic peptides (which have enormous therapeutic potential) and can suppress side reactions such as epimerization during fragment condensations and aspartimide formation. TFA‐stable backbone protecting groups with safety‐catch mechanisms have transformed NCL by comprehensively addressing the aggregation problem in solution. However, there are still opportunities to improve on the current state of the art of Fmoc SPPS and NCL. Further enhancements in protecting group design (e.g., improved acid lability), and their incorporation promise to lead to advances in peptide discovery science, drug development, large‐scale manufacturing, and potentially the synthesis of longer targets such as small enzymes.

## Conflict of Interests

The authors declare no conflict of interest.

## Data Availability

Data sharing is not applicable to this article as no new data were created or analyzed in this study.
